# Functionalized Nitrile Oxides and Their Synthetic Equivalents: Recent Advances in Generation Methods and Synthetic Applications

**DOI:** 10.3390/molecules31142525

**Published:** 2026-07-20

**Authors:** Nagatoshi Nishiwaki

**Affiliations:** School of Engineering Science, Kochi University of Technology, Tosayamada, Kami, Kochi 782-8502, Japan; nishiwaki.nagatoshi@kochi-tech.ac.jp; Tel.: +81-887-531051

**Keywords:** functionalized nitrile oxide, reverse electron demand 1,3-dipolar cycloaddition, synthetic equivalent, isoxazole, oxadiazoles

## Abstract

Functionalized nitrile oxides have attracted increasing attention because the incorporated functional groups not only influence cycloaddition reactivity but also provide valuable handles for subsequent molecular diversification. Despite their considerable synthetic potential, however, the development of practical methods for generating functionalized nitrile oxides has remained challenging because suitable precursors are often difficult to access and many functional groups are incompatible with conventional generation conditions. Consequently, only a limited number of reliable precursor systems have been established. This review summarizes recent advances in the generation of nitrile oxides bearing synthetically valuable acyl, ester, amide, and cyano functionalities, together with the development of synthetic equivalents that circumvent the intrinsic instability of these reactive intermediates. Particular emphasis is placed on 2-methyl-4-nitroisoxazoline-5(2*H*)-one (MeIOx), which serves as a practical precursor to (*N*-methylcarbamoyl)nitrile oxide. Remarkably, this nitrile oxide is generated simply by treatment with water under neutral conditions and undergoes efficient 1,3-dipolar cycloaddition with alkenes, alkynes, nitriles, and 1,3-dicarbonyl compounds to afford structurally diverse isoxazol(in)e and 1,2,4-oxadiazole derivatives. Furthermore, post-cycloaddition transformation of the *N*-methylcarbamoyl group into carboxyl, ester, amide, acyl, and formyl functionalities enables MeIOx to function as a practical synthetic equivalent of a broad range of functionalized nitrile oxides. The review also highlights the unique chemistry of the pyridinium salt PyIOx, whose ring-opening reaction provides cyano-*aci*-nitroacetate as a synthetic equivalent of the highly unstable (cyano)nitrile oxide. These complementary strategies significantly expand the scope of nitrile oxide chemistry and establish practical platforms for the synthesis of highly functionalized heterocycles.

## 1. Introduction

The 1,3-dipolar cycloaddition reaction is one of the most powerful and versatile strategies for constructing five-membered heterocyclic frameworks through the simultaneous formation of two covalent bonds in a single synthetic operation. Since the pioneering work of Huisgen [1], this class of reactions has become a cornerstone of modern synthetic organic chemistry because of its excellent atom economy, high regioselectivity, and broad substrate compatibility.

Among the various 1,3-dipoles, nitrile oxides have attracted particular attention owing to their exceptionally high reactivity toward a wide range of dipolarophiles. They readily undergo cycloaddition reactions with alkynes, alkenes, nitriles, and imines to afford isoxazol(in)e and oxadiazol(in)e derivatives, structural motifs that are frequently encountered in pharmaceuticals, agrochemicals, natural products, and functional materials [2,3,4,5,6,7,8,9,10,11,12,13,14,15]. Owing to the synthetic utility of these heterocyclic frameworks, nitrile oxide cycloaddition has become an indispensable transformation in heterocyclic and medicinal chemistry.

A characteristic feature of nitrile oxides is their intrinsic instability. Because they readily undergo spontaneous dimerization to form furoxans or rearrangement forming isocyanate [16], they are generally generated in situ from suitable precursors immediately before, or in the presence of, the desired dipolarophile. Although stable nitrile oxide was developed, it has not been widely used [17]. In addition, limited availability of the precursors and severe reaction conditions prevented the use of nitrile oxide in organic synthesis. Consequently, considerable effort has been devoted to the development of reliable and efficient methods for their generation, greatly expanding the scope of nitrile oxide chemistry.

**Scheme 1:**
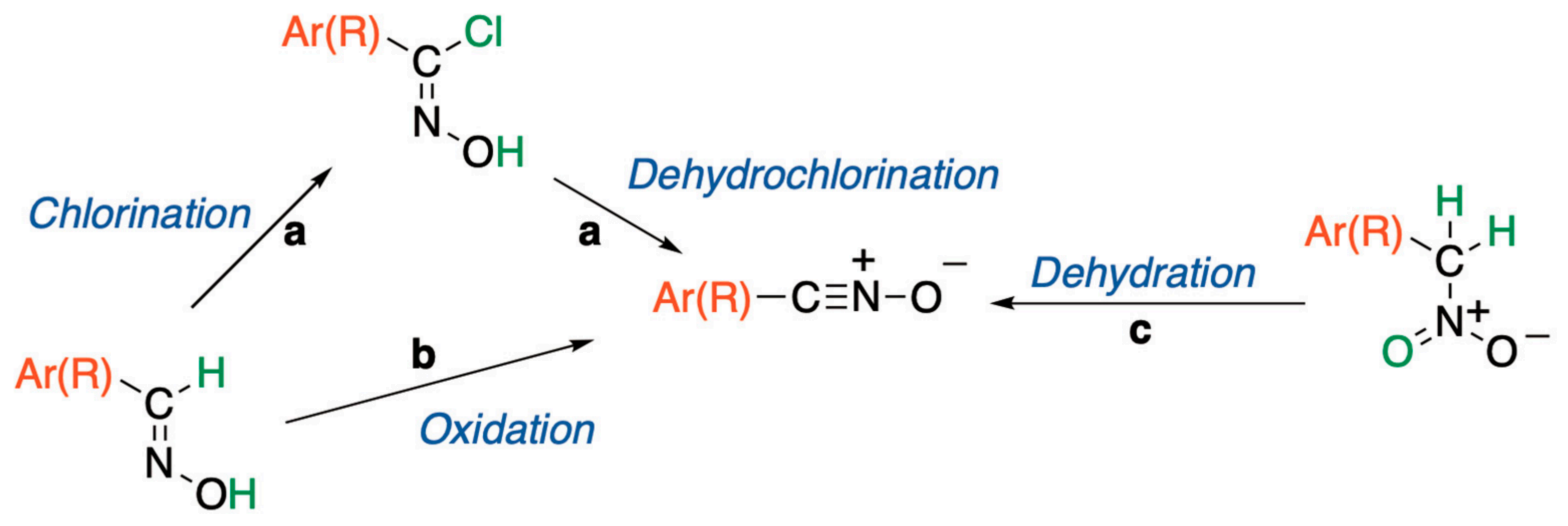
Widely used generation methods of nitrile oxides (a) chlorination of aldoximes followed by dehydrochlorination; (b) oxidation of aldoximes; (c) dehydration of nitro compounds.

Among the available approaches, the most widely employed method involves the base-promoted dehydrochlorination of chloroaldoximes, which are readily prepared by chlorination of aldoximes using reagents such as *N*-chlorosuccinimide (NCS), Cl_2_, SOCl_2_, or PCl_5_ (Scheme 1, route a). Alternatively, nitrile oxides can be generated by the direct oxidation of aldoximes using oxidants including NaOCl, *tert*-BuOCl, NCS, MnO_2_, Pb(OAc)_4_, and hypervalent iodine reagents (Scheme 1, route b). Nitroalkanes also serve as useful precursors and can be converted into nitrile oxides through dehydration with reagents such as Ph-N=C=O, (Ac_2_O), (CF_3_CO)_2_O, and POCl_3_ (Scheme 1, route c).

In addition, transition-metal-mediated methods have further expanded the repertoire of nitrile oxide generation strategies, including those employing Cu(NO_3_)_2_ [18], copper carbenes in combination with *tert*-BuONO [19], and manganese nanoparticles [20]. Transition metals have also been widely utilized to promote nitrile oxide cycloaddition reactions [21,22]. Beyond these approaches, electrochemical oxidation of aldoximes has emerged as an efficient method for nitrile oxide generation [23,24,25], while photocatalytic oxidation has likewise proven to be effective [26]. Furthermore, visible-light-mediated generation of nitrile oxides from hydroxy(imino) acids has recently been reported as a mild and sustainable alternative [27].

The availability of these complementary methodologies has greatly stimulated research on nitrile oxide chemistry, leading to numerous studies on cycloaddition reactions and subsequent transformations of the resulting heterocyclic products [2,3,4,5,6,7,8,9,10,11,12,13,14,15]. Despite these significant advances, however, the overwhelming of majority of reported nitrile oxides bear only simple aryl or alkyl substituents, whereas examples incorporating synthetically versatile functional groups remain relatively scarce.

Although a wide variety of methods for generating nitrile oxides have been developed, examples of nitrile oxides bearing synthetically valuable functional groups remain surprisingly limited. In many cases, the corresponding precursors are not readily accessible, and the desired functional groups are often incompatible with the reaction conditions required for nitrile oxide generation. Consequently, the development of practical, versatile precursors capable of producing functionalized nitrile oxides under mild and operationally simple conditions remains an important challenge in synthetic organic chemistry.

Recent advances have demonstrated that appropriately designed nitrile oxide precursors can not only facilitate the efficient generation of functionalized nitrile oxides but also significantly broaden the synthetic utility of the resulting cycloadducts. In particular, precursors that allow subsequent transformation of the incorporated functional group provide a practical strategy for accessing a diverse range of highly functionalized heterocyclic compounds from a common intermediate.

This review provides an overview of the methodologies developed for the generation of functionalized nitrile oxides, with particular emphasis on their synthetic applications and the limitations associated with existing approaches. Special attention is devoted to 2-methyl-4-nitroisoxazoline-5(2*H*)-one (MeIOx), which has recently emerged as a versatile and practical precursor to (*N*-methylcarbamoyl)nitrile oxide. In addition, the unique reactivity of its pyridinium salt precursor, PyIOx, is discussed as a basis for the development of synthetic equivalents of (cyano)nitrile oxide. Together, these complementary strategies provide efficient access to a broad range of functionalized nitrile oxides and significantly expand the scope of nitrile oxide chemistry in heterocyclic synthesis.

## 2. Functionalized Nitrile Oxides

Functionalized nitrile oxides provide efficient access to highly substituted heterocyclic compounds, whose synthetic utility can be further expanded through subsequent transformation of the incorporated functional groups. Although nitrile oxides have been extensively investigated, relatively few examples of nitrile oxides bearing synthetically useful functional groups have been reported. This limited availability is largely attributed to the difficulty in preparing the corresponding precursors and to poor compatibility of many functional groups with the reaction conditions commonly employed for nitrile oxide generation. This section summarizes the reported methods for generating nitrile oxides bearing synthetically valuable functionalities, including acyl, formyl, carboxy, ester, amide and cyano groups.

### 2.1. Acyl-Substituted Nitrile Oxides

Among functionalized nitrile oxides, (aroyl)nitrile oxides are the most extensively investigated because the aromatic ring effectively stabilizes the nitrile oxide moiety. Consequently, a variety of methods for their generation have been reported. The most common approach is the base-promoted dehydrochlorination of the corresponding chloroaldoxime [28,29,30,31].

**Scheme 2:**
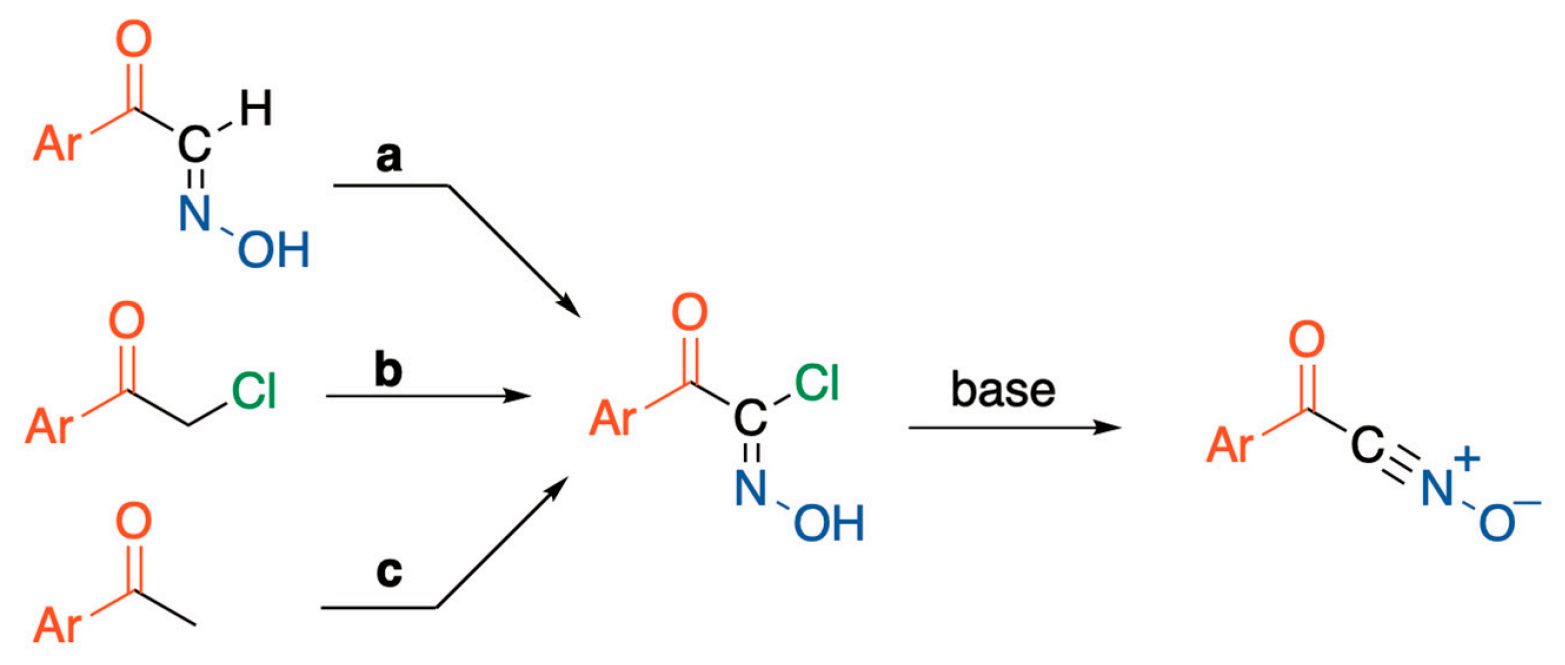
Synthetic routes for chloroaldoxime, precursors to (aroyl)nitrile oxides (a) chlorination of aldoximes; (b) conversion of α-chloroketones to chloroaldoximes; (c) direct transformation of ketones to chloroaldoximes.

Several synthetic routes to aroyl-substituted chloroaldoximes have been developed. These include: (a) chlrorination of the corresponding aldoxime using NCS [32] or HCl/potassium peroxymonosulfate (Oxone) system [33] (Scheme 2, route a); (b) conversion of α-chloroacetophenones into the corresponding chloroaldoximes using HCl and BuONO [34] (Scheme 2, route b); and (c) direct transformation of acetophenone derivatives into chloroaldoximes using NOCl [35], the HCl/HNO_3_/NaNO_2_ system [36], or HCl–*i*-PrONO [37] (Scheme 2, route c). Although these methods provide convenient access to (aroyl)nitrile oxides, their applicability is often limited by the availability of appropriately substituted precursor molecules, particularly when structurally complex or highly functionalized aroyl groups are required.

**Scheme 3:**
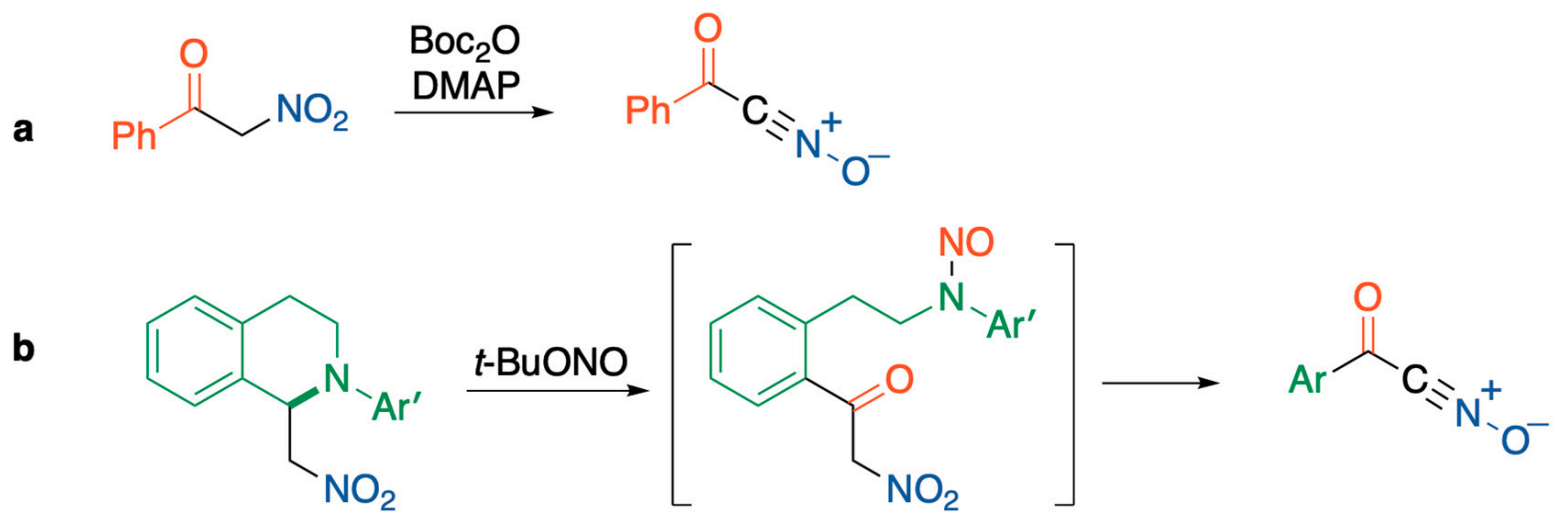
Generation of (aroyl)nitrile oxides from aroyl(nitro)methane (**a**) from benzoyl(nitro)methane; (**b**) from 1-nitromethyl-*N*-aryltetrahydroisoquinolines.

Alternative strategies that avoid the use of chloroaldoximes have also been developed. Tam et al. reported that (benzoyl)nitrile oxide can be generated from benzoyl(nitro)methane by treatment with di-*tert*-butyl dicarbonate (Boc_2_O) in the presence of 4-(dimethylamino)pyridine (DMAP) (Scheme 3a) [38]. Subsequently, Jia et al. expanded this methodology by generating aroyl-substituted nitrile oxides through *tert*-BuONO-induced C–N bond cleavage of 1-nitromethyl-*N*-aryltetrahydroisoquinolines. In this transformation, aroyl(nitro)methanes are formed in situ as key intermediates before conversion into the corresponding nitrile oxides (Scheme 3b) [39,40].

**Scheme 4:**
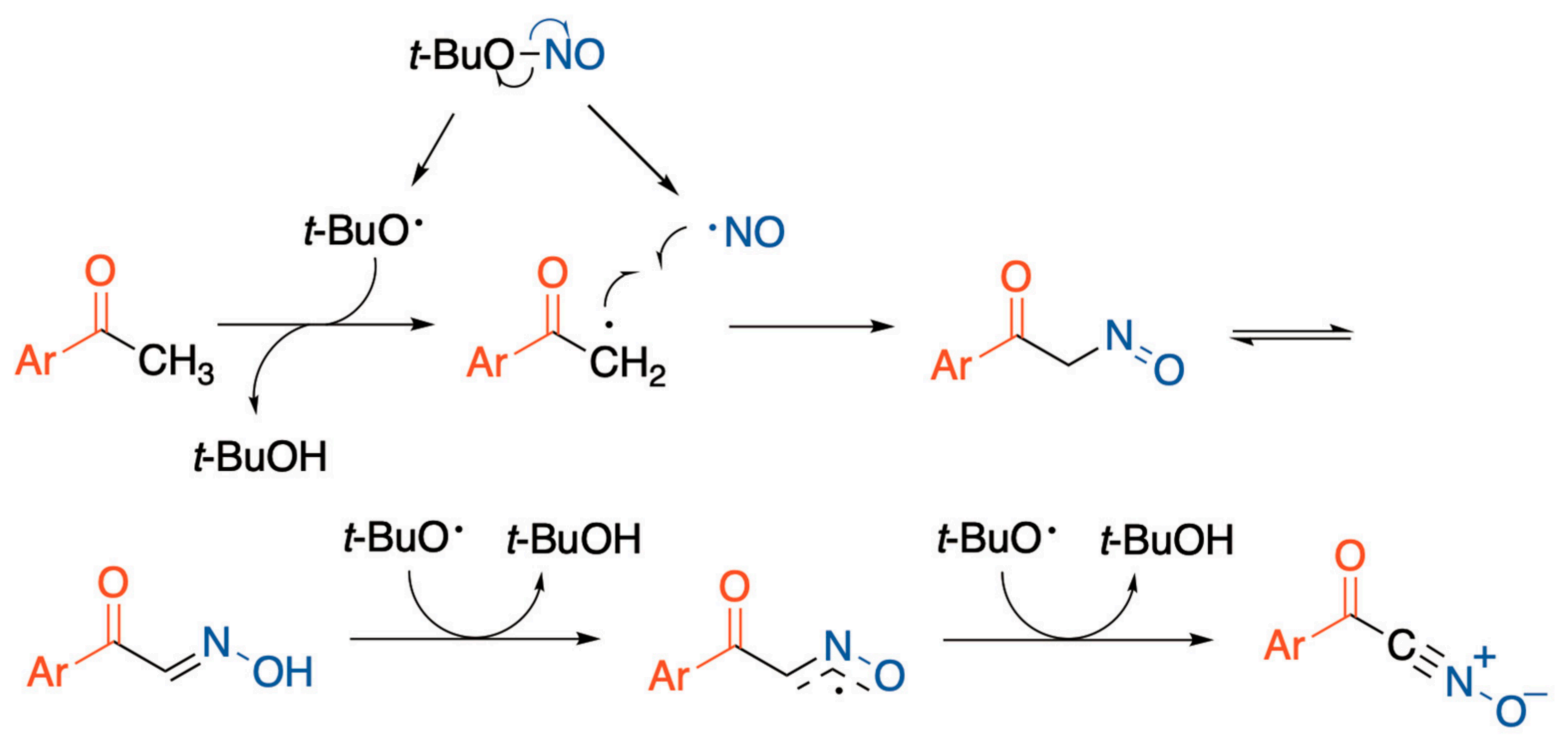
A plausible mechanism for the formation of (aroyl)nitrile oxide from acetophenone derivatives including nitrosoation followed by oxidation.

Recently, Zhang et al. and Sun et al. reported a direct approach starting from acetophenone derivatives [41,42]. In this method, treatment with *tert*-BuONO promotes α-nitrosation followed by oxidative conversion to the corresponding (benzoyl)nitrile oxides (Scheme 4). This strategy eliminates the need for preformed chloroaldoximes and represents a concise alternative for the preparation of and aroyl-substituted nitrile oxides.

In contrast to aroyl-substituted nitrile oxides, considerably fewer examples of (alkanoyl)nitrile oxides have been reported. This limited availability is generally attributed to their own stability and susceptibility of alkanoyl-substituted precursors to the reaction conditions required for nitrile oxide generation.

**Scheme 5:**
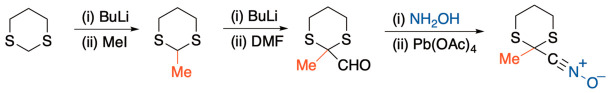
Generation of thioacetal-protected (acetyl)nitrile oxide from dithiane including alkylation, formylation, conversion to oxime, and oxidation.

Among the known examples, (acetyl)nitrile oxide can be generated from the corresponding chloroaldoxime by conventional base-promoted dehydrochlorination [43,44,45]. However, when oxidation of the corresponding oxime with Pb(OAc)_4_ is employed, the acetyl group must first be protected as a thioacetal to prevent undesired side reactions (Scheme 5) [46]. This additional sequence inevitably reduces the overall synthetic efficiency.

An alternative strategy was reported by Roy et al., who achieved the direct generation of (acetyl)nitrile oxide from acetone using ceric ammonium nitrate (CAN) as the oxidant [47]. Although this method eliminates the need for preformed chloroaldoxime intermediates, its substrate scope has thus far remained limited.

**Scheme 6:**
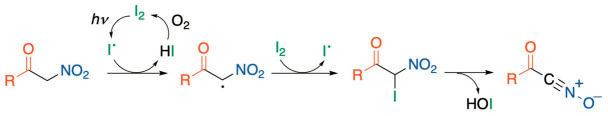
Generation of (acyl)nitrile oxides by photo redox reaction using visible light (440 nm) including α-iodization followed by elimination of HOI.

On the other hand, a method reported by Zhang et al. for the generation of nitrile oxides from methyl ketones enables the introduction of alkanoyl groups, rather than being limited to an acetyl group, into the isoxazole ring [42]. More recently, Giri et al. reported an I_2_-catalyzed photoredox strategy for generating aroyl- and alkanoyl-substituted nitrile oxides from α-nitro carbonyl compounds. One of the proposed reaction mechanisms is shown in Scheme 6 [48].

To date, the generation of nitrile oxides bearing the even more reactive formyl group has not been experimentally realized. Such species have been investigated only in computational studies [49,50], reflecting the substantial challenge associated with generating and handling highly unstable formyl-substituted nitrile oxides under practical reaction conditions.

Overall, although several reliable methods are available for the preparation of aroyl-substituted nitrile oxides, corresponding methodologies for alkanoyl derivatives remain far less developed. Most reported approaches rely on prefunctionalized substrates and often require multistep precursor synthesis or additional protection. Furthermore, their applicability to structurally diverse and highly functionalized substrates remains limited. The development of practical, broadly applicable methods for generating acyl-functionalized nitrile oxides therefore continues to represent an important objective in this field.

### 2.2. Ester-Substituted Nitrile Oxides

**Scheme 7:**
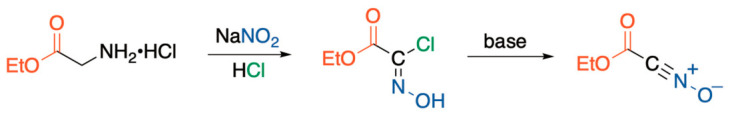
Generation of (ethoxycarbonyl)nitrile oxide from glycine ester via chloroaldoxime followed by dehydrochlorination.

**Scheme 8:**
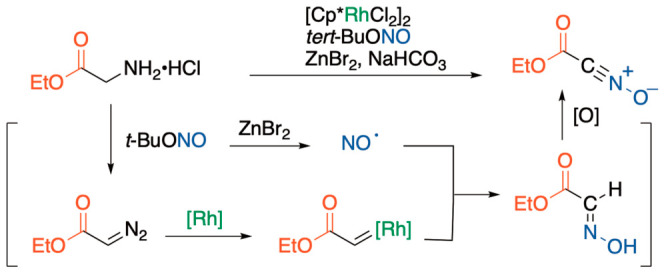
Generation of (ethoxycarbonyl)nitrile oxide from glycine ester including formation of rhodium carbene complex and oxime followed by oxidation (Cp*: cyclopentadienyl ligand).

Whereas carboxy-substituted nitrile oxides have thus far been limited to theoretical investigations [51,52], ester-substituted nitrile oxides are among the most extensively studied functionalized nitrile oxides because of their synthetic utility and the ready availability of suitable precursors. As with most nitrile oxides, they are most commonly generated by the base-promoted dehydrochlorination of the corresponding chloroaldoximes [53,54,55,56,57]. This approach is particularly practical because the requisite chloroaldoximes can be readily prepared from commercially available glycine esters (Scheme 7) [58]. Recently, Hu et al. developed a rhodium-catalyzed oxidation of glycine esters with *tert*-BuONO, in which a rhodium carbene complex is generated in situ (Scheme 8) [59].

**Scheme 9:**
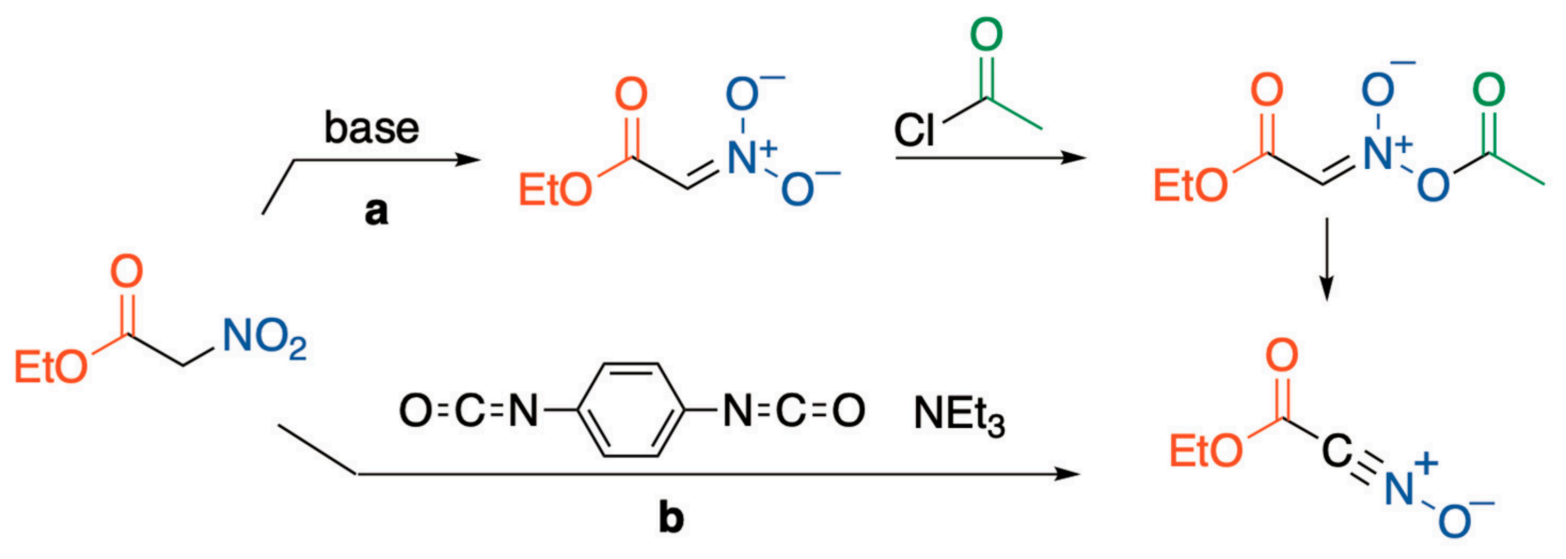
Generation of ester-functionalized nitrile oxide from ethyl nitroacetate using two methods (a) via *O*-acetylated nitronate; (b) Mukaiyama method.

Nitro compounds also serve as versatile precursors for ester-substituted nitrile oxides. In particular, ethyl nitroacetate, a commercially available reagent, provides a convenient entry to these reactive intermediates. Zen et al. [60] and Shimizu et al. [61] independently reported that treatment of ethyl nitroacetate with an acyl chloride generates a mixed nitronic–carboxylic anhydride in situ, which subsequently undergoes dehydration to furnish the corresponding nitrile oxide (Scheme 9, route a). An alternative and widely employed approach is the Mukaiyama method, in which ethyl nitroacetate reacts with an isocyanate to generate the nitrile oxide under mild conditions (Scheme 9, route b) [62]. Owing to the commercial availability of ethyl nitroacetate and the operational simplicity of these procedures, these methods have become standard routes to ester-functionalized nitrile oxides.

**Scheme 10:**
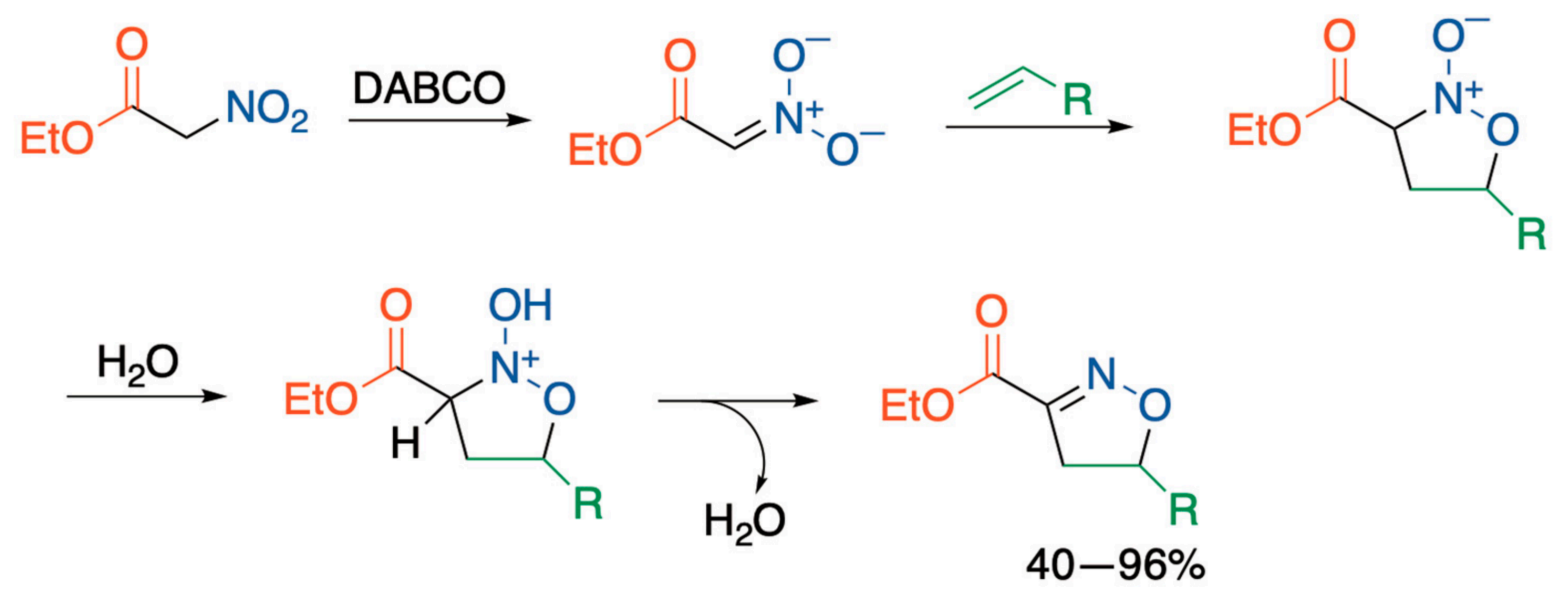
DABCO-induced cycloaddition of ethyl nitroacetate with dipolarophiles including cycloaddition of nitronate followed by dehydration.

In contrast to the generation methods described above, Machetti et al. demonstrated that ethyl nitroacetate itself can function as a synthetic equivalent of an ethoxycarbonyl-substituted nitrile oxide [63,64]. When reactions with suitable dipolarophiles are carried out in the presence of catalytic amounts of 1,4-diazabicyclo [2.2.2]octane (DABCO), cycloadducts are obtained without the intermediacy of a free nitrile oxide. Instead, the nitronate anion directly participates as the 1,3-dipole, providing an efficient alternative strategy for accessing ester-substituted isoxazoline and isoxazole derivatives (Scheme 10).

**Scheme 11:**
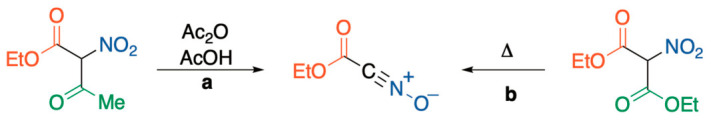
Generation of ester-functionalized nitrile oxide from nitrated 1,3-dicarbonyl compounds (a) acetylation followed by deacetylation; (b) thermolysis of nitrated diethyl malonate.

Nitrated 1,3-dicarbonyl compounds also serve as useful precursors to ester-functionalized nitrile oxides. Representative examples include derivatives of ethyl acetoacetate (Scheme 11, route a) [65] and diethyl malonate (Scheme 11, route b) [66]. Their high reactivity is generally attributed to the enhanced acidity of the α-proton, which facilitates carbonyl-group elimination through a pseudo-intramolecular process [67].

Overall, ester-substituted nitrile oxides are among the most accessible functionalized nitrile oxides, largely because their key precursor, ethyl nitroacetate, is commercially available and can be converted into nitrile oxides by several reliable methods. Nevertheless, the structural diversity of readily available precursors remains limited, making the direct preparation of nitrile oxides bearing modified ester substituents challenging. Consequently, structural diversification is often achieved through post-cycloaddition functional-group transformations rather than by precursor modification. The development of broadly applicable synthetic methods capable of directly introducing structurally diverse ester functionalities therefore remains an important objective in this area.

### 2.3. Amide-Substituted Nitrile Oxides

Compared with ester-substituted nitrile oxides, amide-substituted nitrile oxides are expected to possess greater synthetic versatility because the nitrogen atom of the amide functionality provides an additional site for structural modification. Nevertheless, only a limited number of examples have been reported to date. This scarcity is primarily attributable to the limited availability of suitable precursors and the difficulty associated with their preparation.

**Scheme 12:**
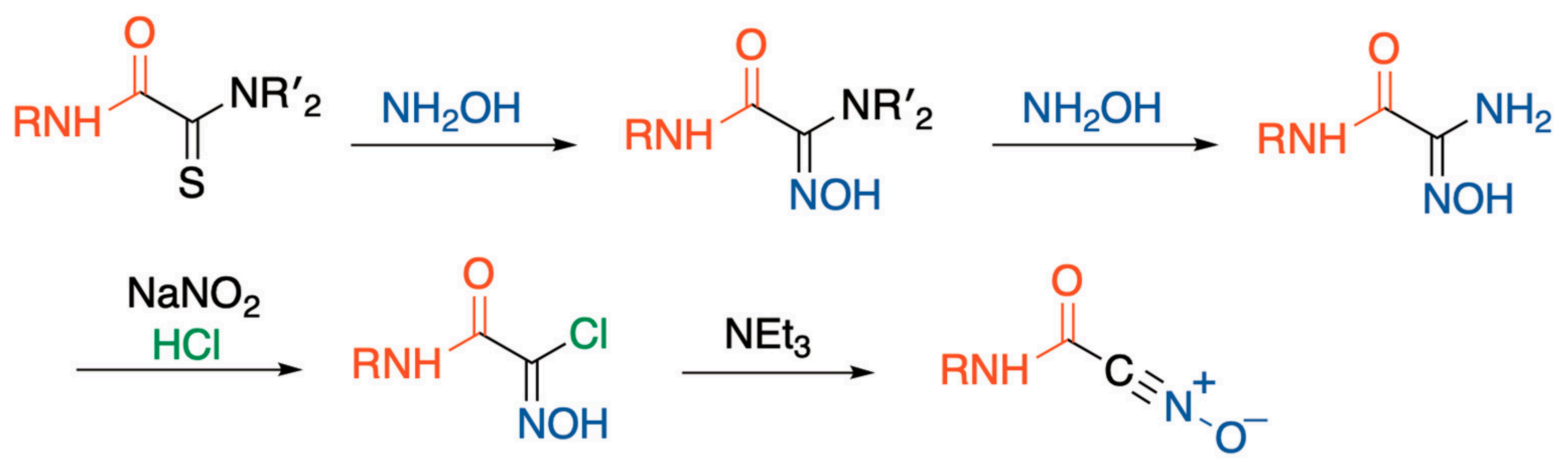
Synthesis of chloroaldoxime and generation of amide-functionalized nitrile oxide by dehydrochlorination.

**Scheme 13:**
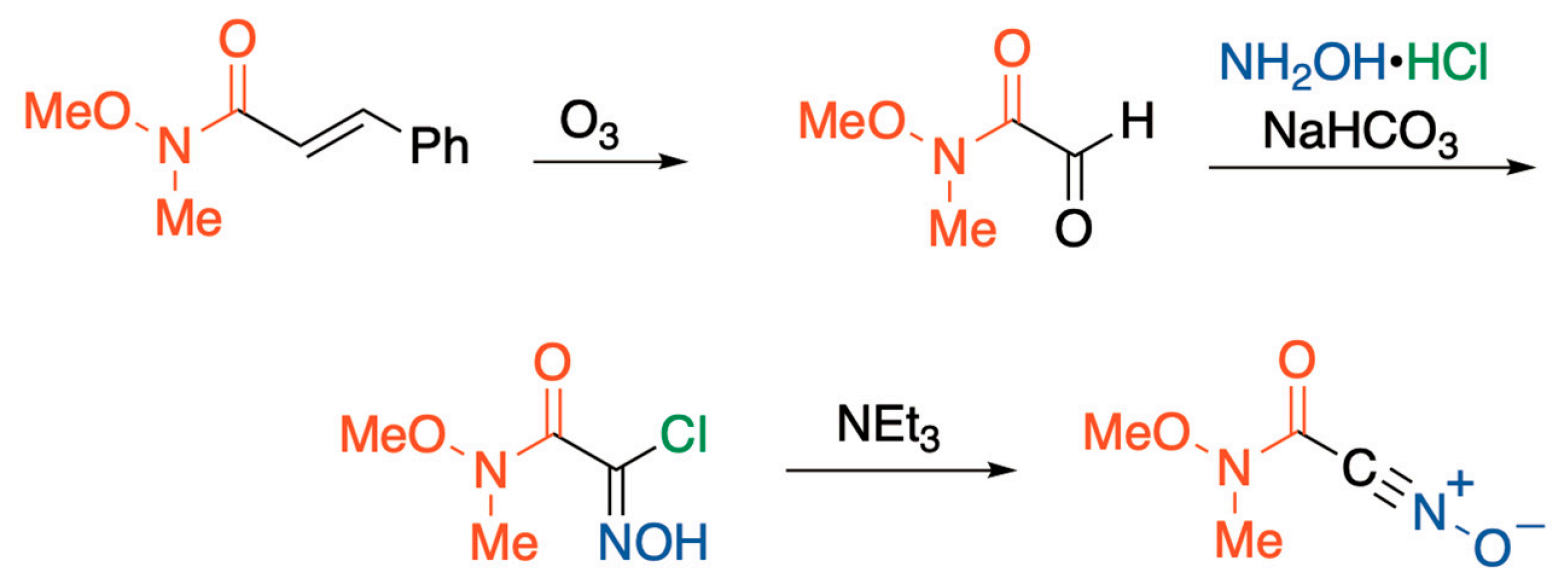
Synthesis of chloroaldoxime possessing Weinreb amide and generation of the corresponding nitrile oxide.

The earliest examples of (carbamoyl)nitrile oxides were generated from the corresponding chloroaldoximes. Yarovenko et al. prepared (carbamoyl)chloroaldoximes by treating monothiooxamides with hydroxylamine, followed by chlorination, and subsequently generated the corresponding nitrile oxides by base-promoted dehydrochlorination (Scheme 12) [68,69]. Franck et al. later reported the preparation of a Weinreb amide-substituted nitrile oxide, starting from cinnamamide via ozonolysis, conversion to the corresponding chloroaldoxime, and subsequent dehydrochlorination (Scheme 13) [70]. In addition, direct oxidation of the corresponding glyoxamide oxime with MnO_2_ enabled the generation of optically active carbamoyl-substituted nitrile oxides, demonstrating that chiral information can be incorporated into the amide substituent [71].

**Scheme 14:**
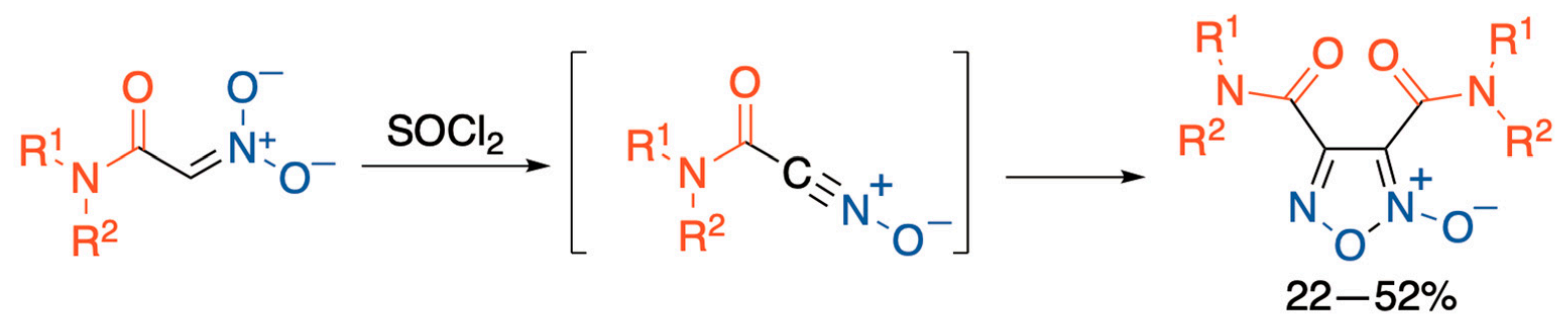
Generation of amide-functionalized nitrile oxide from (nitro)acetamide and its dimerization leading to furoxan.

Alternative methods based on nitroamide precursors have also been developed. Paul et al. first isolated a cycloadduct derived from (*N*-phenylcarbamoyl)nitrile oxide as a minor by-product in the reaction of nitromethane with Ph-N=C=O [72]. Subsequently, Huisgen et al. demonstrated that *N*-phenyl(nitro)acetamide, formed in situ, serves as the actual precursor of the corresponding nitrile oxide [73]. Building on these findings, Joule et al. developed a more practical approach in which nitroacetamides are converted into carbamoyl-substituted nitrile oxides by treatment with SOCl_2_ (Scheme 14) [74]. Blackmond et al. showed Machetti–De Sarlo cycloaddition can be applied to this conversion [75].

**Scheme 15:**
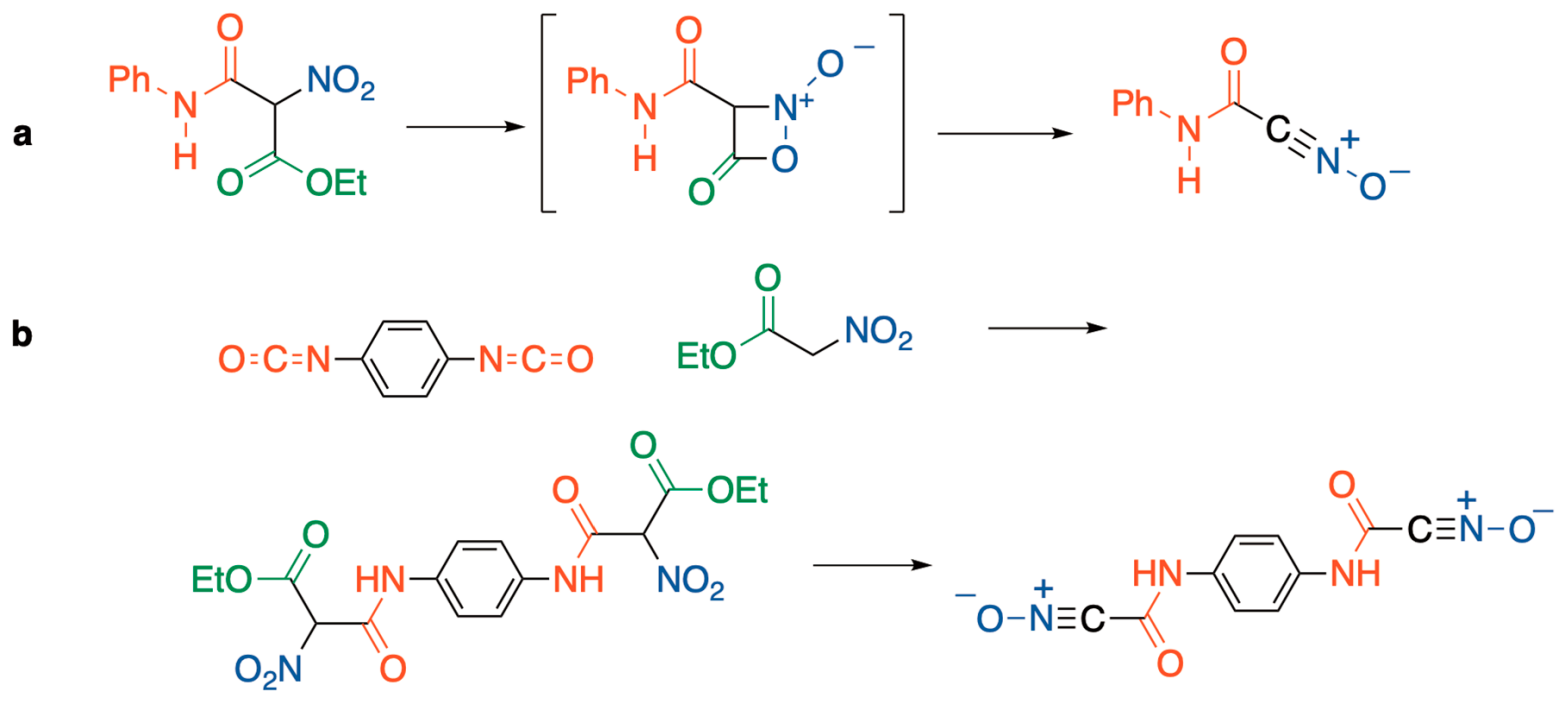
Generation of amide-functionalized nitrile oxides (**a**) by thermolysis of nitrated malonic acid amide ester; (**b**) by Mukaiyama method using nitroacetate.

As discussed in Section 2.2, nitrated 1,3-dicarbonyl compounds also provide useful precursors to functionalized nitrile oxides. Shimizu et al. demonstrated that heating a malonic acid amide ester in refluxing mesitylene (165 °C) generates a carbamoyl-substituted nitrile oxide via a transient oxazetidine intermediate (Scheme 15, route a) [61]. Furthermore, when bis(malonic acid derivatives) are employed, the corresponding bis(nitrile oxides) are generated in situ and have been successfully utilized as cross-linking agents for polymer synthesis (Scheme 15, route b) [76,77].

Overall, the chemistry of carbamoyl-substituted nitrile oxides remains considerably less developed than that of their acyl- and ester-substituted counterparts. The principal limitation is the lack of readily accessible and versatile precursor molecules. Because the amide nitrogen can accommodate a wide variety of substituents, carbamoyl-substituted nitrile oxides offer exceptional opportunities for structural diversification and downstream functionalization. The development of practical and broadly applicable precursor systems capable of generating structurally diverse carbamoyl-substituted nitrile oxides therefore represents an important challenge and a promising direction for future research.

### 2.4. Cyano-Substituted Nitrile Oxides

**Scheme 16:**
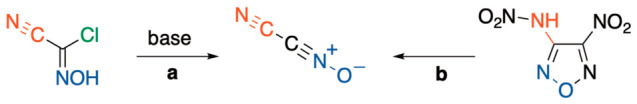
Generation of nitrile oxide possessing a cyano group (a) dehydrochlorination of cyano-substituted chloroaldoxime; (b) by thermolysis of furazan derivatives.

Among functionalized nitrile oxides, (cyano)nitrile oxide (cyanogen *N*-oxide) is one of the most challenging species to generate and utilize because of its extreme instability. To date, only a limited number of preparation methods have been reported. Conventional approaches include the base-promoted dehydrochlorination of chloro(cyano)aldoxime (Scheme 16, route a) [78,79] and flash vacuum pyrolysis of furazan derivatives (Scheme 16, route b) [80].

Despite the successful generation of (cyano)nitrile oxide by these methods, its practical application in synthetic organic chemistry remains extremely limited. The nitrile oxide undergoes rapid decomposition under the reaction conditions, and suitable precursor molecules are not readily accessible. Consequently, although (cyano)nitrile oxide has occasionally been employed as a model substrate in theoretical studies [49], its use as a general synthetic reagent has not been realized.

These limitations have prompted the search for alternative strategies that circumvent the direct generation of this highly unstable nitrile oxide. In particular, the development of synthetic equivalents of (cyano)nitrile oxide, capable of reproducing its characteristic cycloaddition reactivity under mild and practical conditions, has emerged as an important objective. Such approaches are expected to substantially broaden the synthetic utility of cyano-substituted heterocyclic compounds.

## 3. Nitroisoxazolones

### 3.1. Synthesis of Nitroisoxazolones

**Scheme 17:**
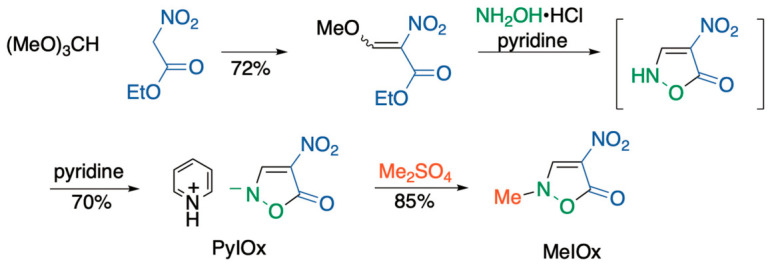
Synthesis of PyIOx from ethyl nitroacetate by successive condensation with trimethyl orthoformate and hudroxylamine. Subsequent methylation leading to MeIOx.

2-Methyl-4-nitroisoxazoline-5(2*H*)-one (MeIOx) is readily prepared in three steps from commercially available ethyl nitroacetate (Scheme 17) [81]. Condensation of ethyl nitroacetate and trimethyl orthoformate affords α-nitro-β-(methoxy)acrylate, which undergoes cyclization upon treatment with hydroxylamine hydrochloride to furnish the nitroisoxazolone framework. Because the initially formed nitroisoxazolone possesses a highly acidic N–H proton, it is readily isolated as the corresponding pyridinium salt (PyIOx), which precipitates directly from the reaction mixture as pale-yellow needles. Subsequent methylation of PyIOx with dimethyl sulfate provides MeIOx in good yield. Although this synthetic sequence efficiently affords the methyl derivative, attempts to introduce alkyl substituents other than methyl have thus far been unsuccessful.

Both PyIOx and MeIOx possess several synthetically valuable structural features, including a nitroenamine moiety, an α,β-unsaturated lactone, and an N–O bond, each of which can participate in characteristic transformations. Owing to this unique combination of reactive functionalities, nitroisoxazolones exhibit remarkably versatile reactivity and serve as valuable building blocks for the synthesis of a broad range of highly functionalized organic molecules. As described in the following sections, these compounds also function as practical precursors to functionalized nitrile oxides and their synthetic equivalents, thereby providing access to transformations that are difficult to achieve using conventional nitrile oxide chemistry.

### 3.2. Generation of Carbamoylnitrile Oxide

**Scheme 18:**
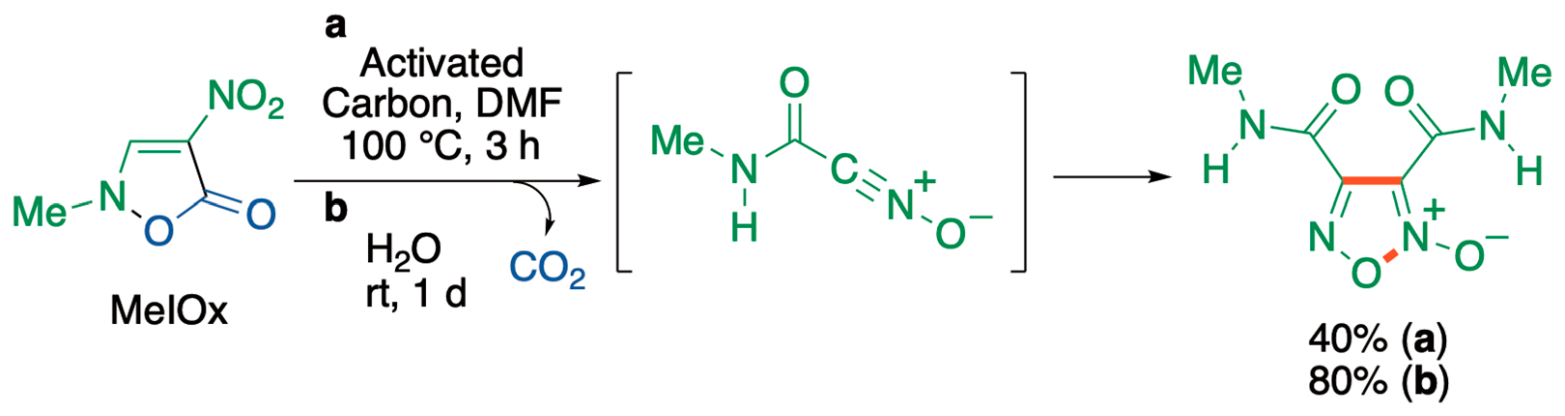
Generation of (carbamoyl)nitrile oxide from MeIOx (a) by addition of activated carbon in DMF; (b) by stirring in water. The red solid lines mean the newly formed bonds between two molecules of nitrile oxides.

The first indication that MeIOx could serve as a precursor to (carbamoyl)nitrile oxide was reported by Ariga et al. [82]. They found that the addition of activated carbon to a hot *N*,*N*-dimethylformamide (DMF) solution of MeIOx induced vigorous gas evolution, which was attributed to decarboxylation. Under these conditions, the corresponding furoxan was isolated in 40% yield (Scheme 18, route a), providing clear evidence that (carbamoyl)nitrile oxide had been generated in situ and subsequently underwent dimerization.

Subsequently, Nishiwaki et al. demonstrated that water plays a crucial role in the generation of (carbamoyl)nitrile oxide [83]. Remarkably, simply stirring MeIOx in water at room temperature for 24 h afforded the corresponding furoxan in 80% yield (Scheme 18, route b), indicating that the nitrile oxide can be generated efficiently under exceptionally mild and operationally simple conditions without the need for catalysts, bases, or other activating reagents. This finding represents a significant advantage over conventional methods for nitrile oxide generation, which generally require specially designed precursors and relatively harsh reaction conditions.

**Scheme 19:**
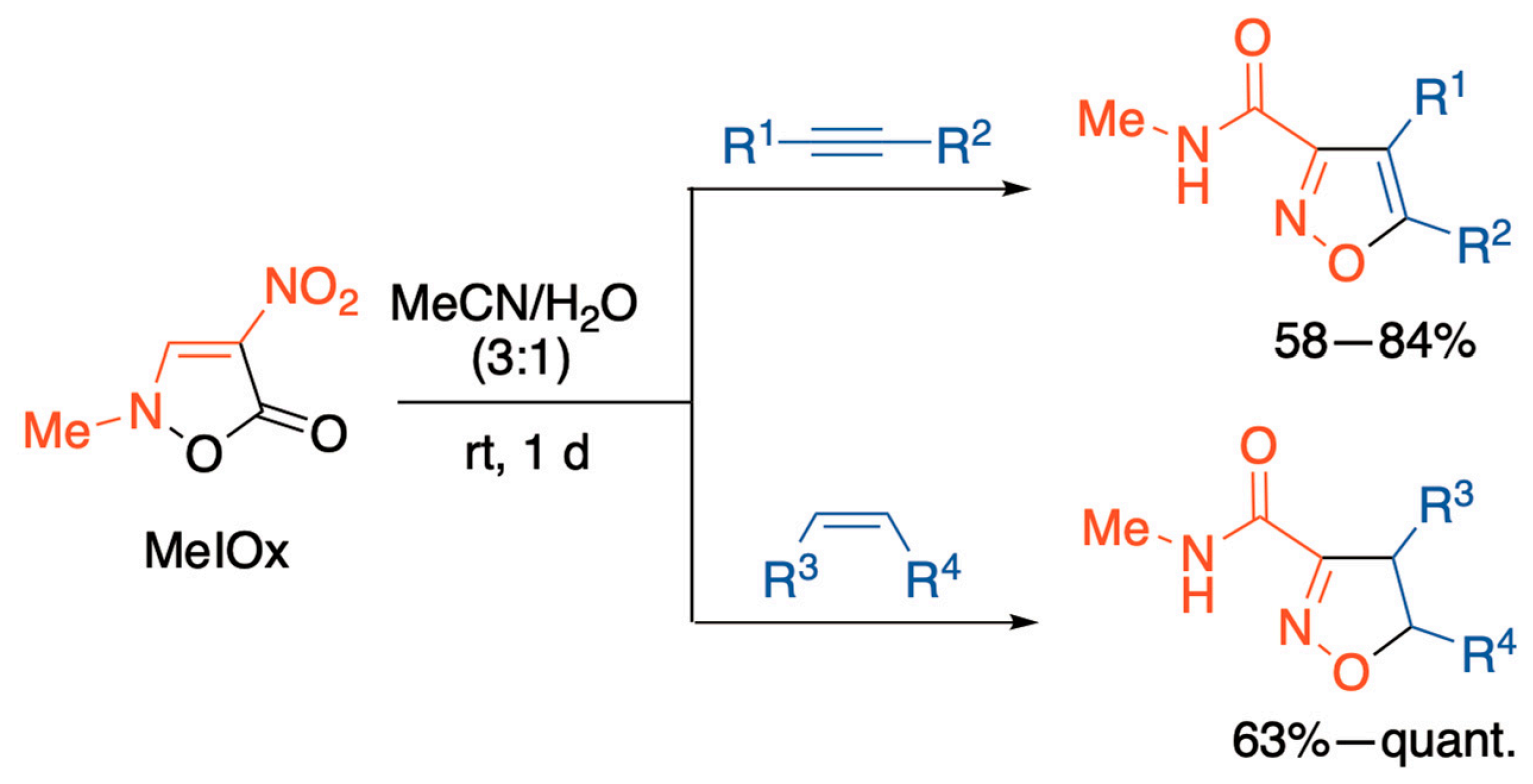
Synthesis of functionalized isoxazol(in)es.

When the nitrile oxide is generated in the presence of suitable dipolarophiles, it is efficiently trapped by 1,3-dipolar cycloaddition to afford the corresponding heterocyclic products (Scheme 19) [83]. Alkynes furnish isoxazoles, whereas alkenes afford the corresponding isoxazolines. A mixed solvent system of MeCN/H_2_O (3:1, *v*/*v*) was found to provide optimal results because it effectively dissolves both MeIOx and a wide range of dipolarophiles, while no additional catalyst or additive is required.

For terminal alkynes, cycloaddition proceeds with excellent regioselectivity to afford 5-substituted isoxazoles (R^1^ = H; R^2^ = Ph, CH_2_OH, or CH_2_Br). Electron-deficient alkynes, such as diethyl acetylenedicarboxylate (R^1^ = R^2^ = COOEt), also participate smoothly in the reaction to give highly functionalized tricarbonyl-substituted isoxazoles. Likewise, cycloaddition with alkenes exhibits broad substrate compatibility. Both electron-rich alkenes (R^3^ = H; R^4^ = Ph, *n*-Pr, CH_2_OH, CH_2_OEt, or OEt) and electron-poor alkenes (R^3^ = H; R^4^ = COOEt or COMe) undergo regioselective cycloaddition to furnish the corresponding isoxazolines in good yields.

These results clearly demonstrate that MeIOx functions as a practical and versatile precursor to (carbamoyl)nitrile oxide. Unlike conventional precursor systems, which often require multistep synthesis and harsh activation conditions, MeIOx generates the nitrile oxide under remarkably mild aqueous conditions while exhibiting broad compatibility with a variety of dipolarophiles. This unique reactivity forms the basis for the synthetic applications described in the following sections.

### 3.3. Mechanism of (Carbamoyl)nitrile Oxide Formation

**Scheme 20:**
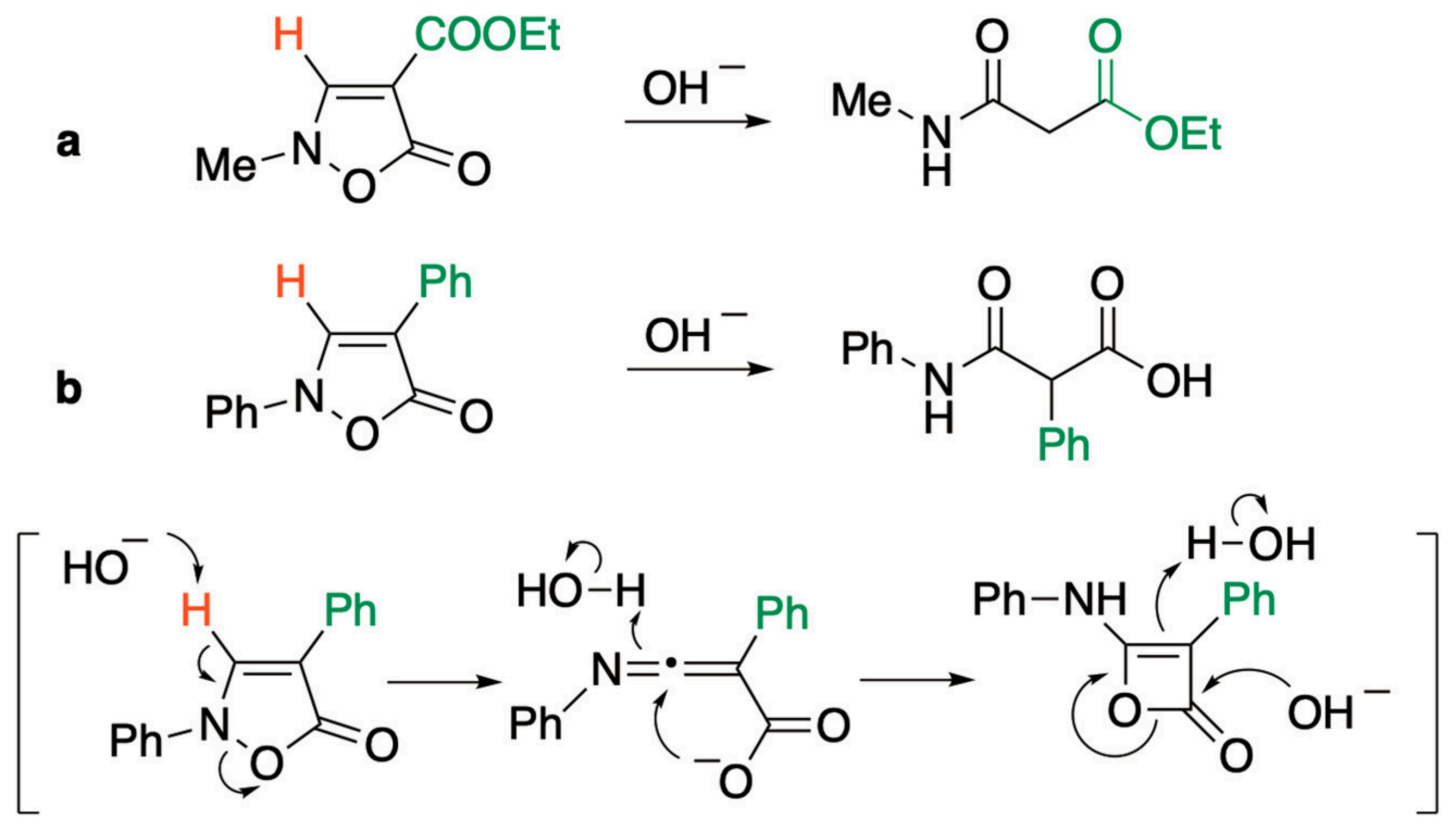
Base-induced ring opening of isoxazolones (**a**) of 4-ethoxycarbonyl derivative; (**b**) of 4-phenyl derivative.

The generation of (carbamoyl)nitrile oxide from MeIOx is considered to be initiated by ring opening of the isoxazolone framework. Insight into this process can be obtained from earlier studies on structurally related isoxazolones. Ulrich et al. reported that a 4-ester-substituted isoxazolone undergoes base-promoted ring opening to afford a malonic acid amide ester (Scheme 20, route a) [84]. Subsequently, Woodman et al. demonstrated that analogous ring opening also occurs for 4-phenylisoxazolones (Scheme 20, route b) and proposed a mechanism involving a transient oxetane intermediate [85,86].

These observations suggest that MeIOx should undergo ring opening even more readily, because the strongly electron-withdrawing nitro group stabilizes the developing negative charge more effectively than an ester substituent. Furthermore, the combined resonance-withdrawing effects of the nitro and carbonyl groups, together with the inductive effects of the nitrogen and oxygen atoms within the isoxazolone, substantially enhance the acidity of the proton at the C3 position, facilitating deprotonation under remarkably mild conditions.

**Scheme 21:**
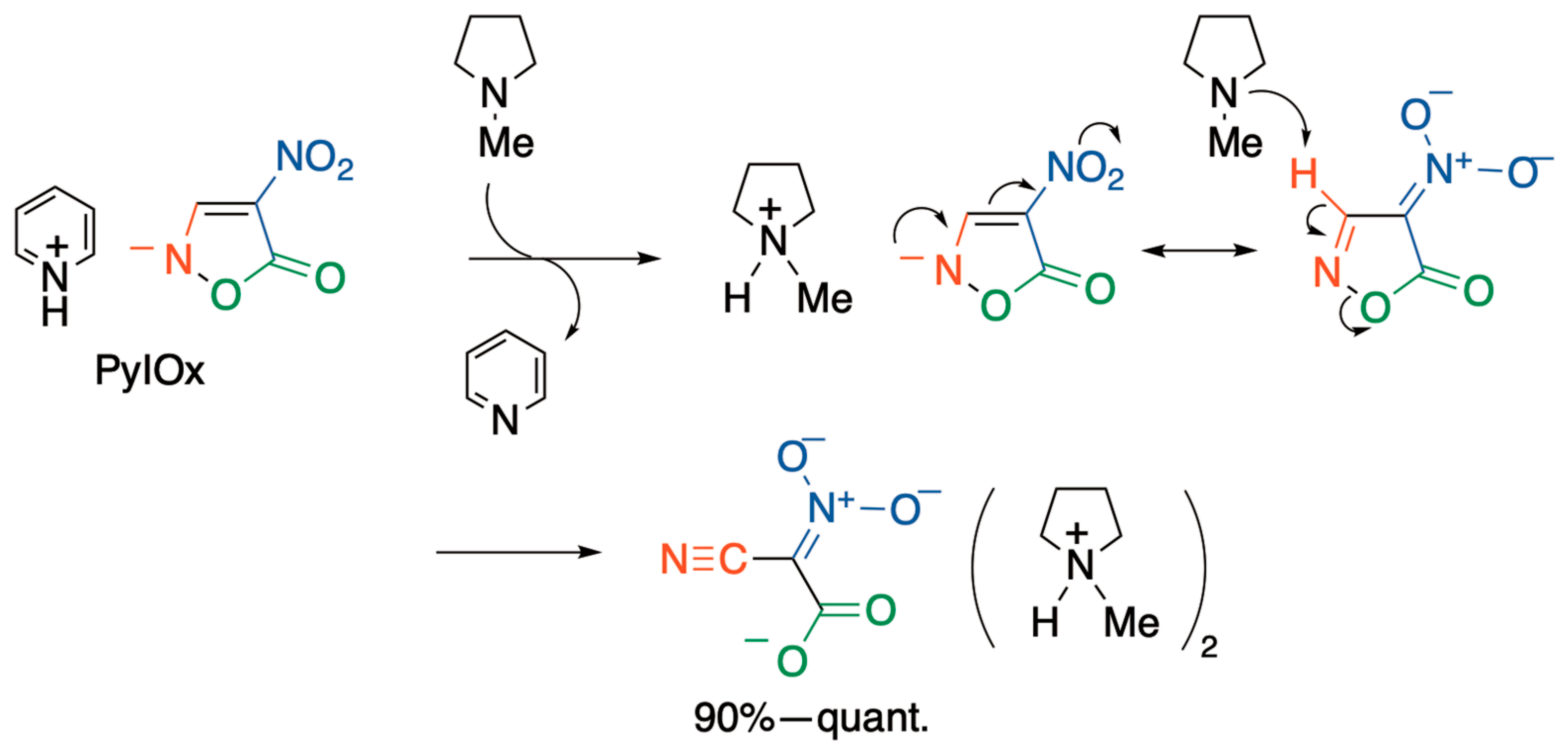
Ring opening reaction of PyIOx leading to cyano-*aci*-nitroacetate.

Experimental evidence supporting this proposal was obtained from studies on PyIOx. Despite its anionic nature, PyIOx readily undergoes ring opening upon treatment with bases such as hydroxide, carbonate, or *N*-methylpyrrolidine [87]. The reaction requires two equivalents of base. Following cation exchange with the first equivalent, deprotonation at the C3 position by the second base triggers cleavage of the N–O bond, affording dianionic cyano-*aci*-nitroacetate (Scheme 21). This highly reactive intermediate subsequently serves as a versatile synthon for the construction of a variety of polyfunctionalized molecular architectures [88].

**Scheme 22:**
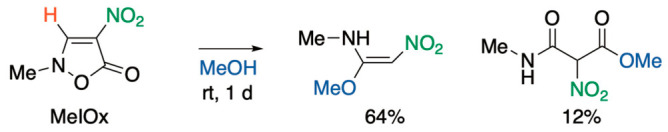
Reaction of MeIOx with methanol.

Considering the pronounced susceptibility of anionic PyIOx toward ring opening, the electronically neutral MeIOx is expected to undergo the same transformation in the presence of even weaker bases. Consistent with this expectation, stirring MeIOx in methanol at room temperature affords nitroenamine together with (nitro)malonic acid amide ester (Scheme 22) [85]. The formation of these products closely parallels the reactivity observed for the phenylisoxazolone shown in Scheme 20, route b and strongly suggests that methanol itself acts as a Brønsted base, abstracting the proton at the C3 position. By analogy, water is considered to play the same role during the generation of (carbamoyl)nitrile oxide under aqueous conditions.

**Scheme 23:**
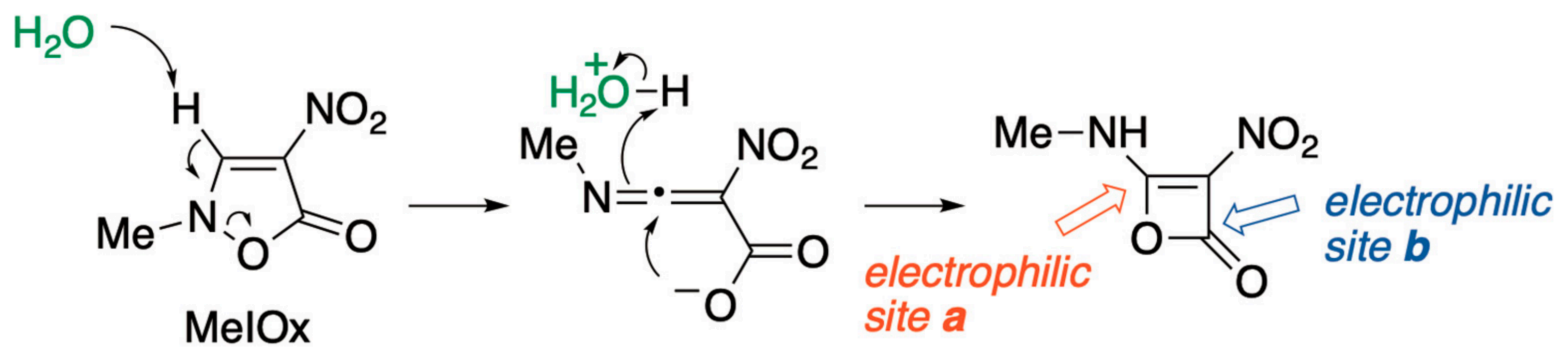
A plausible mechanism for generation of oxetane intermediate.

**Scheme 24:**
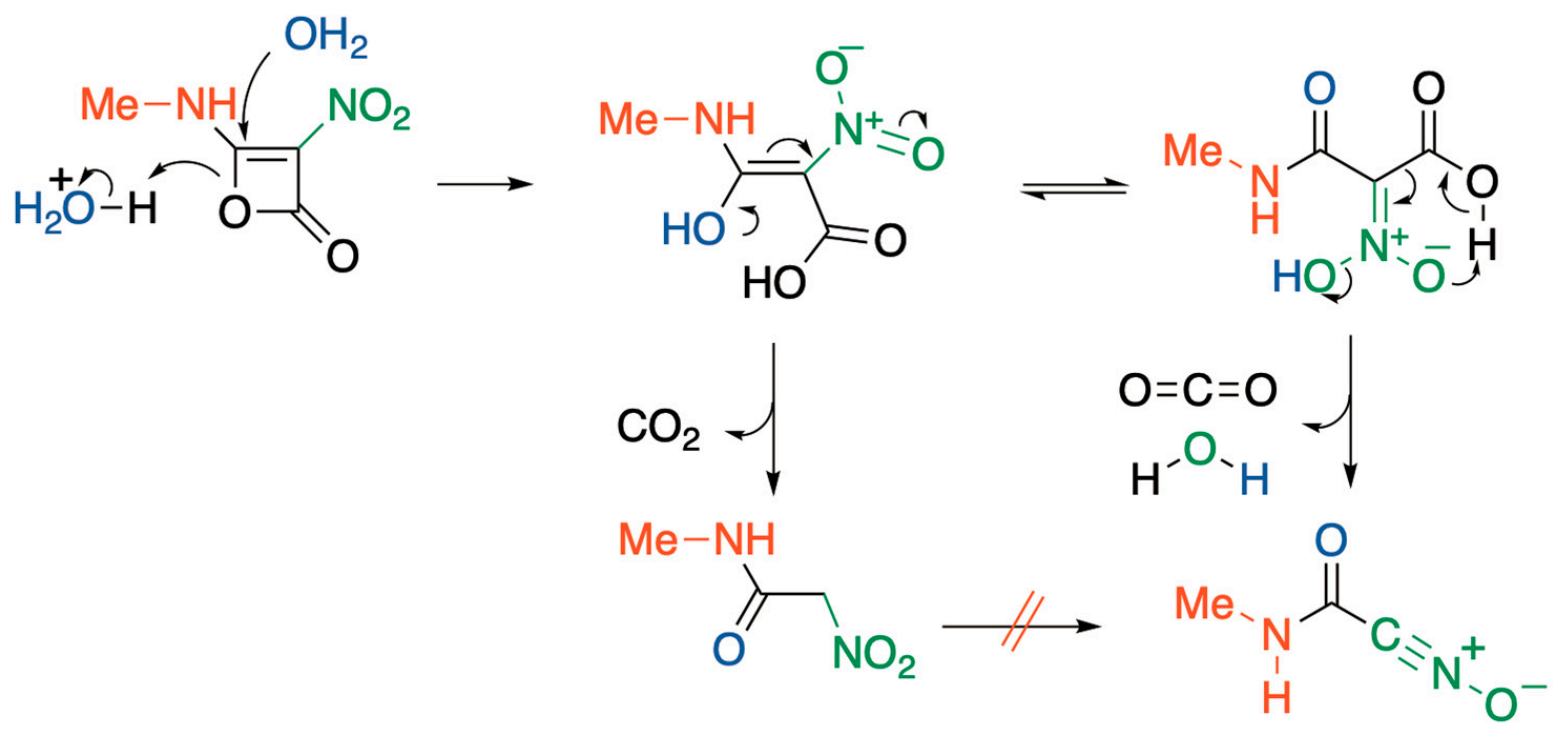
A plausible mechanism for generation of (carbamoyl)nitrile oxide including attack of water at site a.

**Scheme 25:**
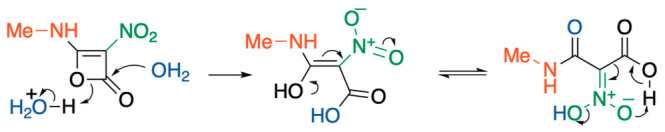
A plausible mechanism for generation of (carbamoyl)nitrile oxide including attack of water at site b.

A plausible mechanism for the generation of (carbamoyl)nitrile oxide from MeIOx is proposed in Scheme 23, Scheme 24 and Scheme 25 [83]. The reaction is initiated by deprotonation at the C3 position by water, which acts as a Brønsted base. Subsequent cleavage of the N–O bond affords [(nitroketene)imine]carboxylate, a highly reactive intermediate. In accordance with the mechanism proposed by Woodman et al. [86], the nucleophilic carboxylate then intramolecularly attacks the electrophilic central carbon atom of the cumulene moiety, leading to the formation of a strained oxetane intermediate (Scheme 23).

The resulting oxetane possesses two electrophilic carbon atoms that are susceptible to nucleophilic attack. Insight into their relative reactivity can be obtained from the methanolysis experiment shown in Scheme 22. Methanol attacks either site a or site b, affording nitroenamine and (nitro)malonic acid amide ester, respectively. Because nitroenamine is formed preferentially, site a is considered to be the more electrophilic center. A similar preference is expected for the corresponding reaction with water.

When water attacks site a, ring opening of the oxetane affords an enol intermediate, which undergoes tautomerization to produce a nitronic acid via the corresponding (nitro)malonic acid amide (Scheme 24). The nitronic acid is then converted into (carbamoyl)nitrile oxide through a concerted decarboxylation–dehydration process. An alternative stepwise pathway involving sequential decarboxylation followed by dehydration appears much less likely. As discussed in Section 2.2 (Scheme 7 and Scheme 8), dehydration of nitro compounds generally requires specific activating conditions for nitrile oxide formation. Consistent with this observation, *N*-methyl(nitro)acetamide is sufficiently stable to be isolated and does not spontaneously generate the corresponding nitrile oxide. In contrast, the nitronic acid intermediate proposed in Scheme 24 is expected to undergo rapid decarboxylation and dehydration, most likely facilitated by intramolecular hydrogen bonding, thereby enabling direct formation of (carbamoyl)nitrile oxide.

Attack of water at site b initially follows a different bond-cleavage pathway but ultimately converges on the same (nitro)malonic acid-derived intermediates. Consequently, both reaction pathways lead to the identical nitronic acid intermediate and ultimately furnish (carbamoyl)nitrile oxide (Scheme 25).

Overall, the proposed mechanism rationally explains the exceptionally mild generation of (carbamoyl)nitrile oxide from MeIOx under neutral aqueous conditions. The cooperative effects of facile ring opening, intramolecular cyclization through an oxetane intermediate, and concerted decarboxylation–dehydration distinguish this process from conventional nitrile oxide generation methods and account for the unique reactivity of MeIOx as a practical nitrile oxide precursor.

### 3.4. Cycloaddition with Nitriles

**Figure 1:**
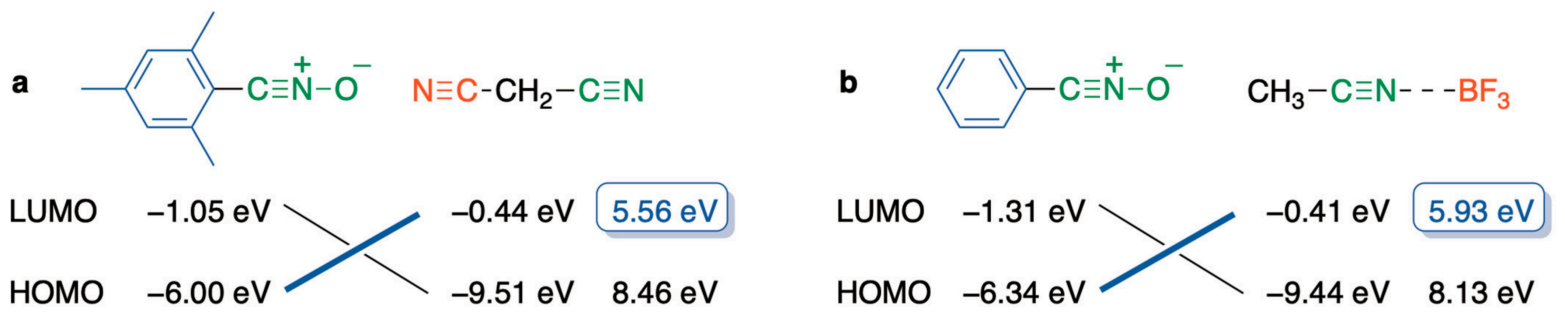
Calculated energy correlations between nitrile oxides and nitriles using B3LYP 6-31G* (**a**) cycloaddition of (aryl)nitrile oxide and electron-deficient nitrile; (**b**) cycloaddition of (aryl)nitrile oxide with aliphatic nitrile in the presence of Lewis acid. Solid lines mean energetically advantageous combinations.

The 1,3-dipolar cycloaddition of nitrile oxides with nitriles provides one of the most straightforward approaches to the synthesis of 1,2,4-oxadiazoles, and numerous synthetic methods have been reported [2,89]. In most cases, however, the nitrile oxide component is limited to relatively stable benzonitrile oxides or their derivatives bearing electron-donating substituents, such as alkyl, alkoxy, and hydroxy groups. Consequently, the substituent at the 3-position of the oxadiazole is generally restricted to an aryl group [90,91]. Conversely, nitriles bearing electron-withdrawing substituents are frequently employed as dipolarophiles because of their enhanced electrophilicity [92,93]. Even when aliphatic nitriles are used, activation with a Lewis acid is generally required to reduce the electron density of the nitrile carbon and promote cycloaddition [94,95]. These substrate requirements are consistent with the conventional normal electron-demand pathway, in which the reaction proceeds through interaction between the HOMO of the nitrile oxide and the LUMO of the nitrile (Figure 1a,b) [96].

**Scheme 26:**
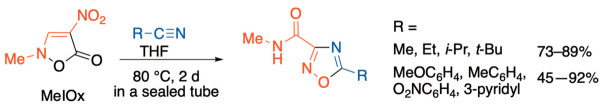
Synthesis of functionalized oxadiazoles.

**Figure 2:**
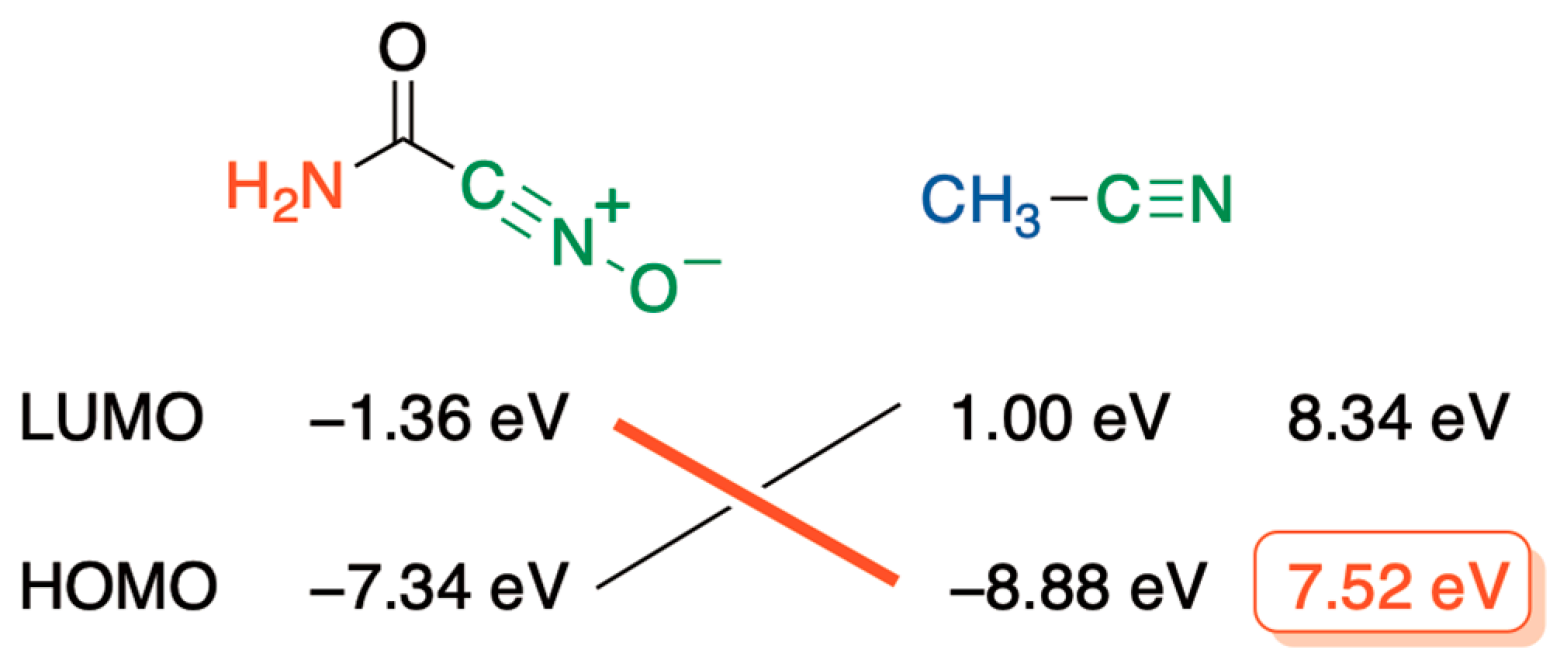
Calculation results indicating inverse electron-demand 1,3-dipolar cycloaddition. Solid line means energetically advantageous combination.

In contrast to these conventional systems, MeIOx exhibits a markedly different reactivity profile [97]. When MeIOx is allowed to react with nitriles in the presence of tetrahydrofuran (THF) as a base, the in situ-generated (carbamoyl)nitrile oxide undergoes smooth cycloaddition with both aromatic and aliphatic nitriles to afford the corresponding 1,2,4-oxadiazoles (Scheme 26). Notably, aliphatic nitriles participate efficiently without Lewis acid activation, representing a significant departure from previously reported methodologies. Computational analysis revealed that this unusual reactivity arises because the cycloaddition preferentially proceeds through an inverse electron-demand pathway involving interaction between the LUMO of the nitrile oxide and the HOMO of the nitrile (Figure 2), rather than the conventional orbital interaction observed for most nitrile oxide cycloadditions [96].

**Figure 3:**
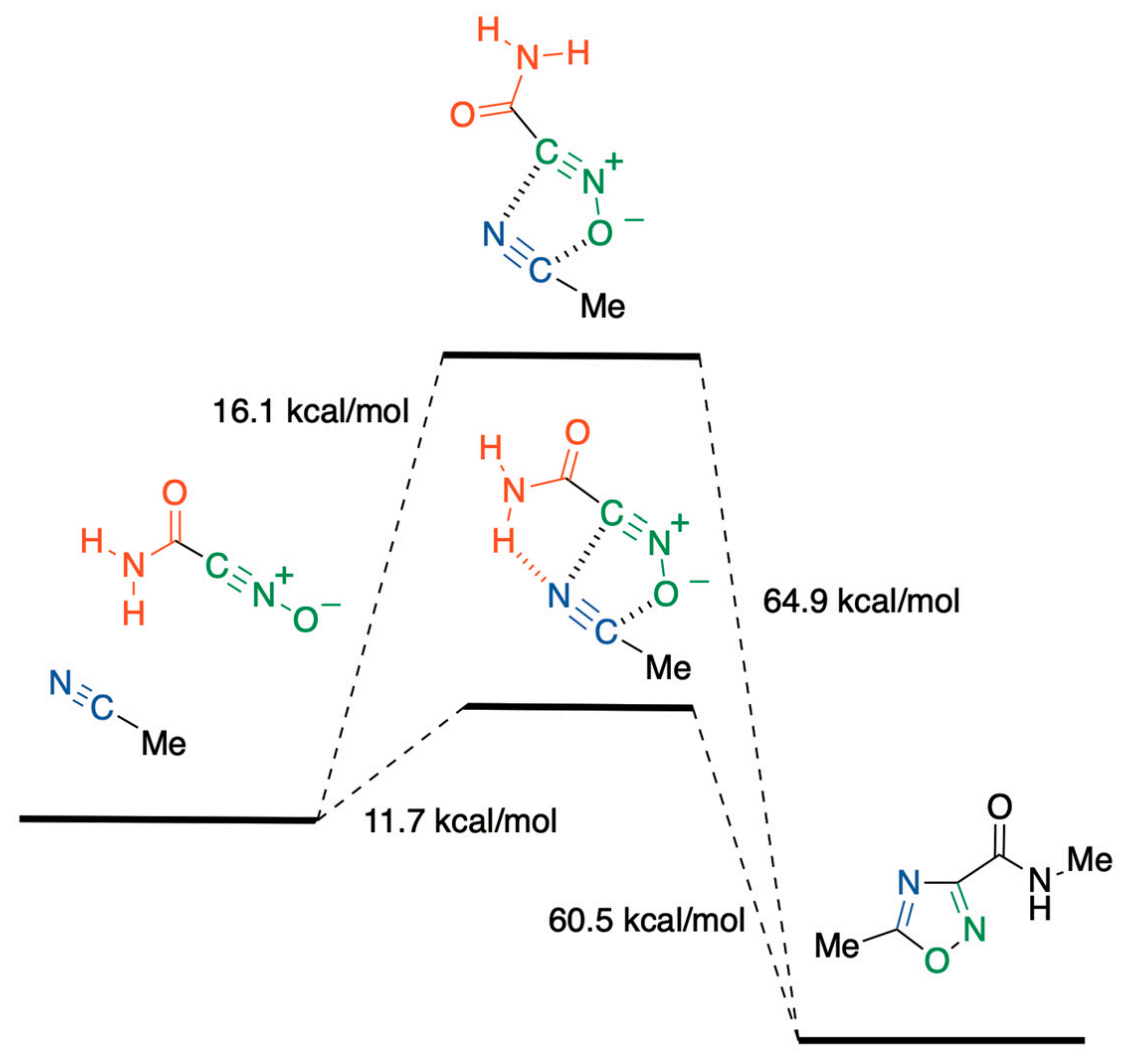
Activation energies for two kinds of transition states.

An additional factor contributing to this unique reactivity appears to be the carbamoyl functionality of the nitrile oxide. To the best of our knowledge, cycloaddition reactions of ester-substituted nitrile oxides with nitriles have not been reported. The principal structural difference between carbamoyl and ester substituents is the ability of the former to act as a hydrogen-bond donor. Corsaro et al. demonstrated that hydrogen-bonding interactions can effectively activate nitriles toward cycloaddition [97,98]. By analogy, the carbamoyl group of (carbamoyl)nitrile oxide is considered to activate the nitrile substrate through intermolecular hydrogen bonding. This proposal is supported by computational studies, which show that formation of the hydrogen-bonded complex lowers the activation barrier for cycloaddition (Figure 3). These findings indicate that the carbamoyl group not only serves as a synthetically versatile functional handle but also plays a direct mechanistic role in promoting cycloaddition reactivity.

### 3.5. Cycloaddition with 1,3-Dicarbonyl Compounds

Although 1,3-dicarbonyl compounds represent attractive dipolarophiles because of their versatile synthetic utility, relatively few examples of their cycloaddition with nitrile oxides have been reported. Umesha et al. described the synthesis of 4-acetyl-3-arylisoxazoles through the cycloaddition of isolable aromatic nitrile oxides with acetylacetone [99]. Suzuki et al. subsequently developed a cyclocondensation of sterically hindered aromatic nitrile oxides with cyclic β-diketones [100] and successfully applied this methodology to the synthesis of natural products and biologically active molecules [101]. Other approaches employ preformed sodium enolates of β-keto esters or β-keto amides as dipolarophiles, allowing efficient cycloaddition with nitrile oxides [102,103]. Machetti et al. also reported a base- and copper-catalyzed condensation of primary nitro compounds with various active methylene compounds, including acetylacetone, benzoylacetone, and ethyl acetoacetate [104]. As these reactions are conducted under basic conditions, the scope of substrates is limited. The formation of the corresponding furoxans confirmed the in situ generation of benzoyl- and ester-substituted nitrile oxides. Nevertheless, these reactions generally require strongly basic conditions to generate both the nitrile oxide and the enolate, thereby restricting the substrate scope and increasing the likelihood of competing side reactions.

**Scheme 27:**
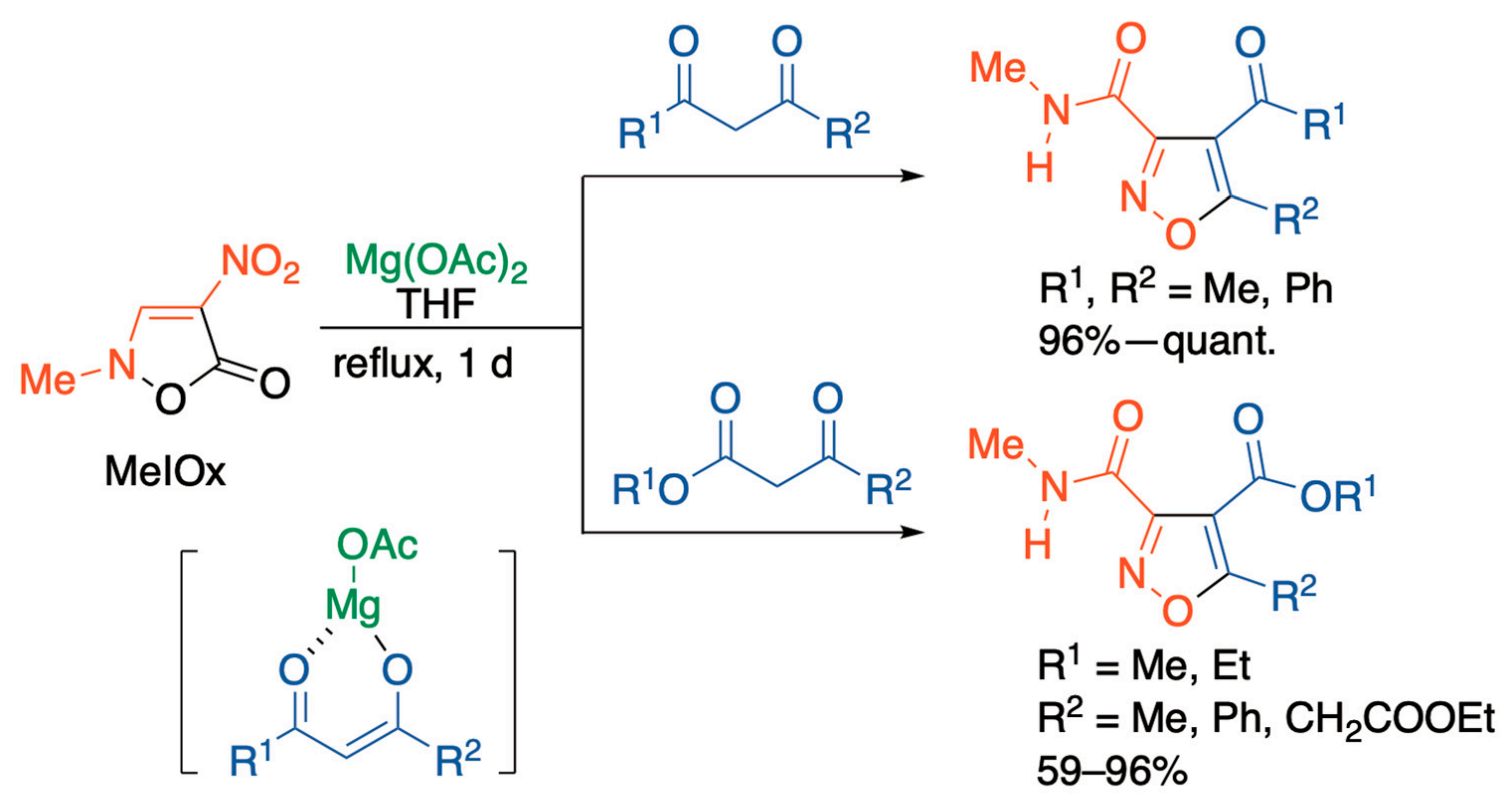
Synthesis of isoxazoles possessing different carbonyl functionalities.

In contrast, MeIOx generates (carbamoyl)nitrile oxide under exceptionally mild conditions using only weak bases such as water or THF [105]. Consequently, the cycloaddition can be performed under nearly neutral conditions, minimizing undesired side reactions and, importantly, permitting the use of Lewis acids that would be incompatible with conventional nitrile oxide generation methods. Among the metal salts examined, Mg(OAc)_2_ proved to be the most effective, presumably because it activates the 1,3-dicarbonyl compound through bidentate chelation while simultaneously stabilizing its enol form. Under these conditions, a broad range of 1,3-dicarbonyl compounds undergoes smooth cycloaddition with the in situ-generated (carbamoyl)nitrile oxide to afford the corresponding heterocyclic products in good yields (Scheme 27) [105].

Electron-deficient substrates generally exhibit lower enol content and therefore reduced reactivity under the neutral conditions. For example, ethyl trifluoroacetoacetate did not readily undergo cycloaddition unless it was first converted into the corresponding sodium enolate, highlighting the importance of sufficient enolization for efficient cycloaddition.

These results demonstrate another distinctive advantage of MeIOx as a nitrile oxide precursor. Because the nitrile oxide can be generated under nearly neutral conditions, catalytic activation of the dipolarophile by Lewis acids becomes feasible, greatly expanding the range of compatible substrates. This strategy provides access to transformations that are difficult to achieve using conventional nitrile oxide generation methods and further illustrates the unique synthetic potential of carbamoyl-substituted nitrile oxides.

### 3.6. Conversion to Other Functional Groups

**Scheme 28:**
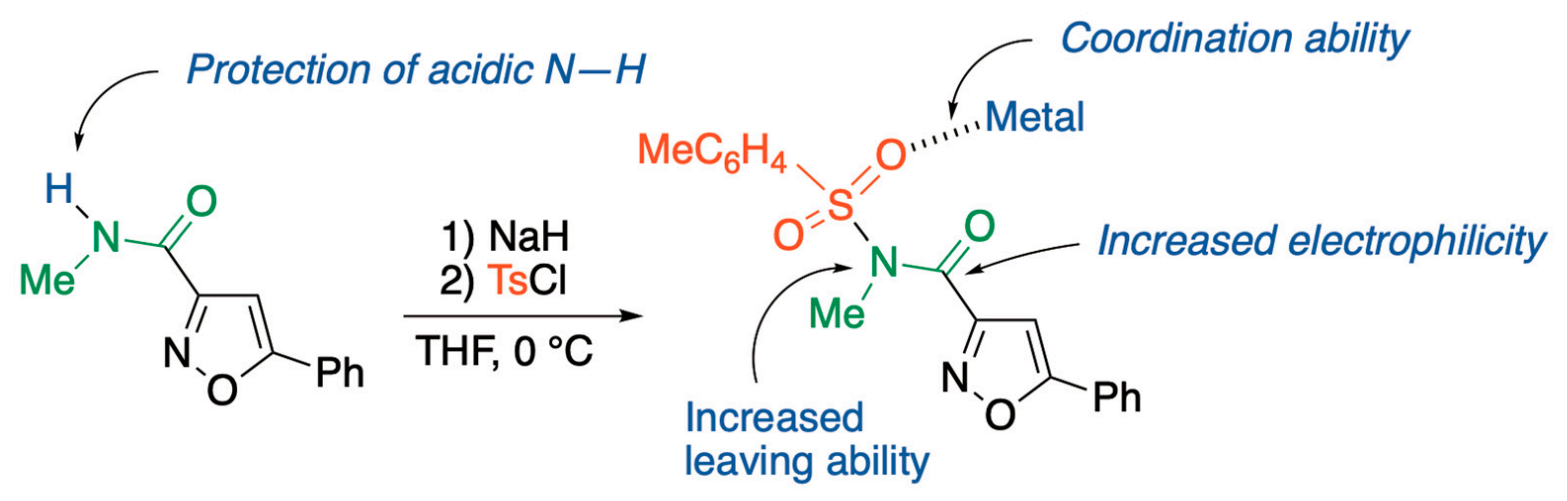
Tosylation of the *N*-methylcarbamoyl group.

Although MeIOx serves as an efficient precursor to (*N*-methylcarbamoyl)nitrile oxide, its synthetic utility is inherently limited by the fixed *N*-methyl substituent on the carbamoyl group. This limitation can be effectively overcome by tosylation of the amide nitrogen, which transforms the carbamoyl group into a versatile platform for subsequent functional-group interconversion (Scheme 28) [106].

Introduction of the tosyl group provides several important advantages. First, it protects the acidic N–H proton of the carbamoyl functionality. Second, its strong electron-withdrawing character enhances the electrophilicity of the amide carbonyl carbon. Third, the resulting tosylamide functions as an excellent leaving group, facilitating nucleophilic substitution. Finally, the tosyl and carbonyl groups cooperate to form a bidentate coordination site for metal ions. Collectively, these features greatly expand the synthetic versatility of the original *N*-methylcarbamoyl functionality.

**Scheme 29:**
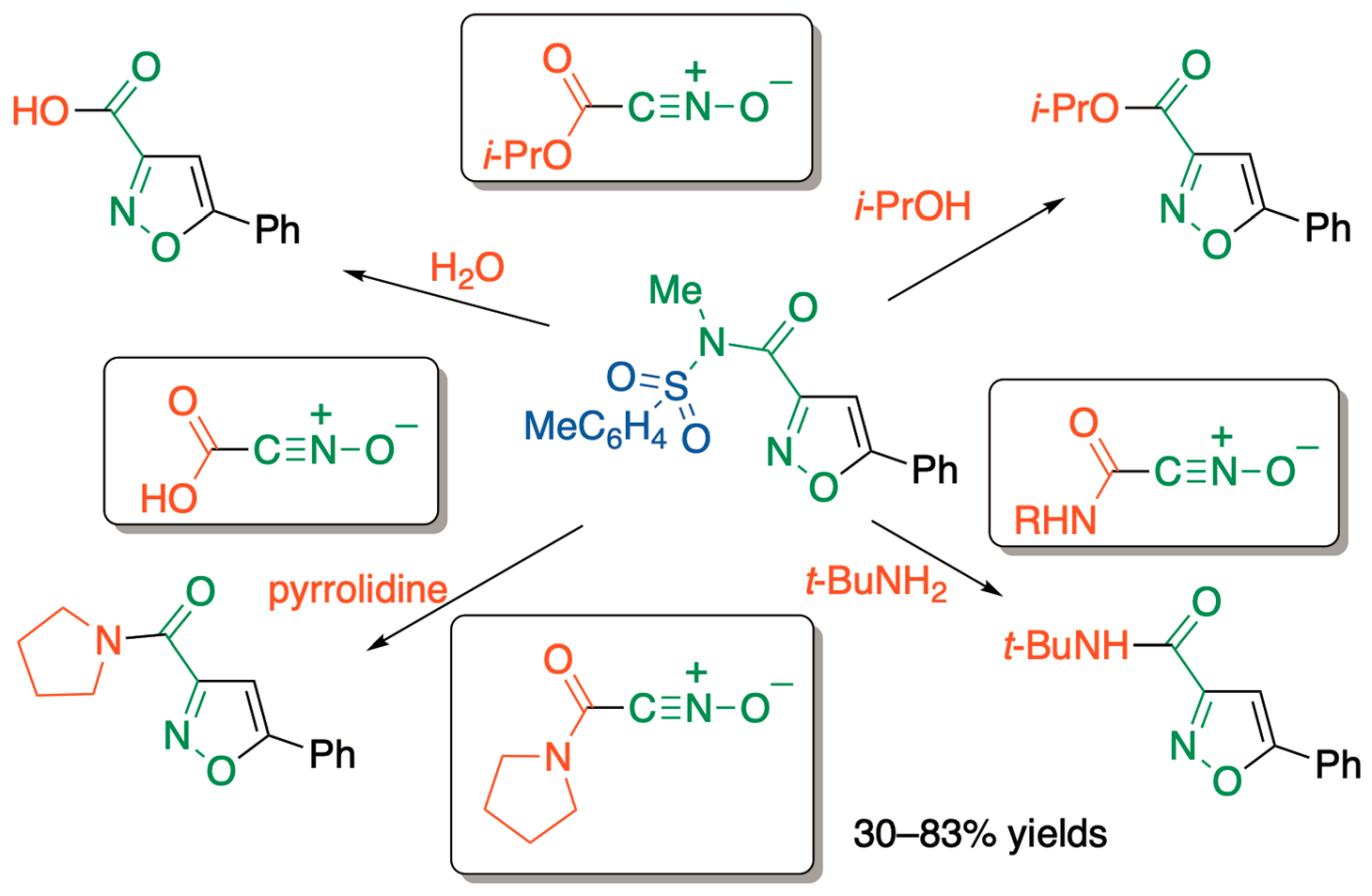
Reactions of *N*-tosylated cycloadduct with hetero nucleophiles.

As a consequence, a variety of functional-group interconversions become possible. Treatment of the tosylated cycloadduct with oxygen nucleophiles, such as water or alcohols, affords the corresponding carboxylic acids and esters, respectively. These products can be regarded as the cycloadducts that would be obtained from carboxy- and ester-substituted nitrile oxides. Likewise, reactions with primary amines, including *n*-propylamine, *sec*-butylamine, and *tert*-butylamine, provide the corresponding *N*-alkylamides, whereas reaction with the secondary amine pyrrolidine furnishes the corresponding tertiary amide (Scheme 29) [106]. Thus, the carbamoyl substituent can be diversified without modifying the nitrile oxide precursor itself.

**Scheme 30:**
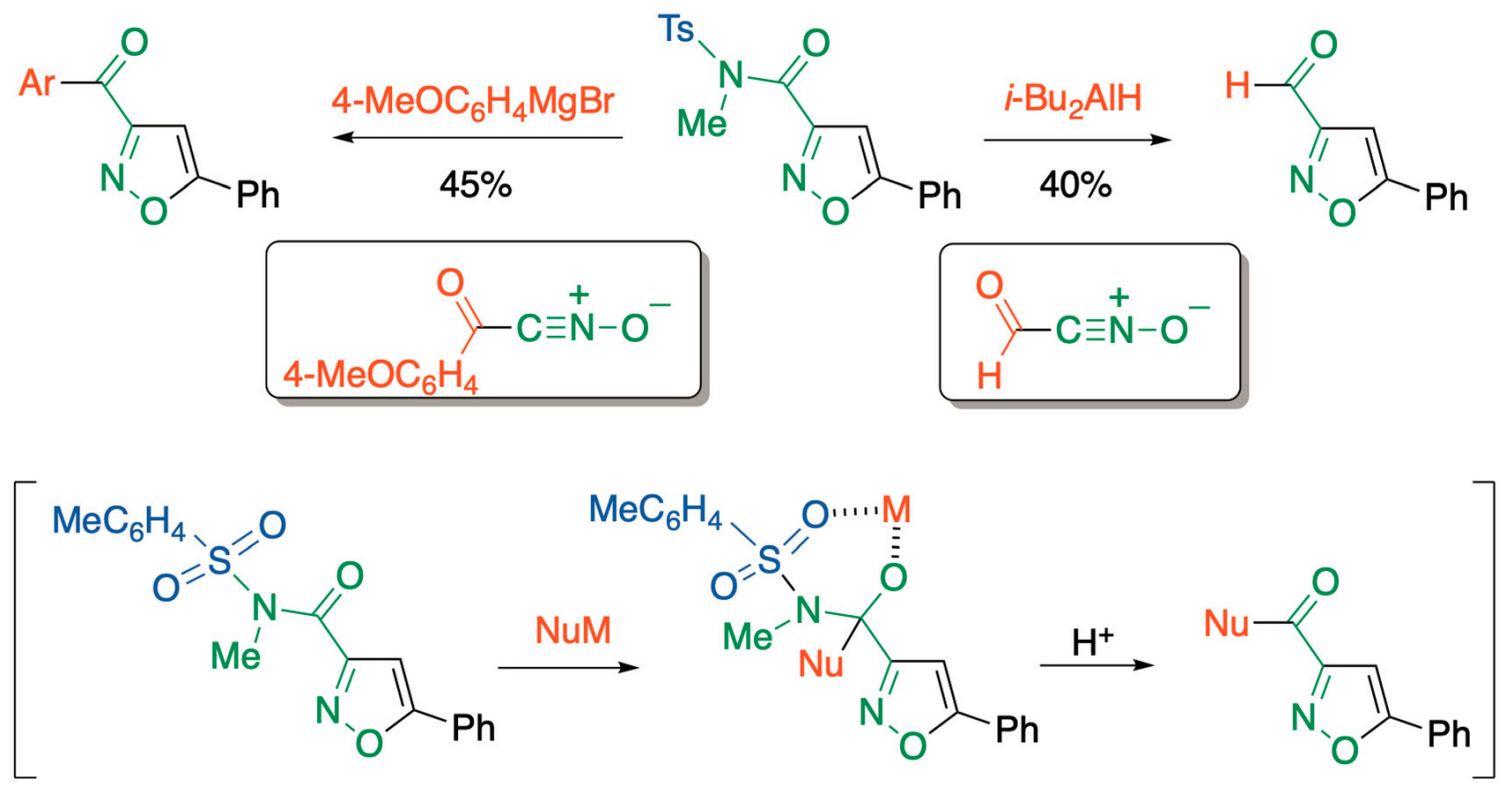
Conversion of amide to aldehyde and ketone using Weinreb amide-like behavior.

The tosylated amide can also be converted into a ketone and aldehyde by treatment with a Grignard reagent and diisobutylaluminum hydride (DIBAL-H), respectively (Scheme 30) [106]. This unique reactivity is attributed to chelation of the metal center by the tosyl and carbonyl oxygen atoms, which stabilizes the tetrahedral intermediate in a manner analogous to that observed for Weinreb amides. Accordingly, these products may be viewed as the cycloadducts derived from acyl- and formyl-substituted nitrile oxides [106].

Overall, tosylation transforms the initially formed *N*-methylcarbamoyl group into a versatile synthetic handle that can be converted into carboxylic acid, esters, amides, ketone, and aldehyde. Consequently, although MeIOx formally generates only (*N*-methylcarbamoyl)nitrile oxide, it effectively functions as a universal synthetic equivalent of a wide range of functionalized nitrile oxides. This strategy significantly expands the scope of functionalized nitrile oxide chemistry while avoiding the need to prepare numerous structurally diverse and often inaccessible nitrile oxide precursors.

### 3.7. Synthetic Equivalent of (Cyano)nitrile Oxide

**Scheme 31:**
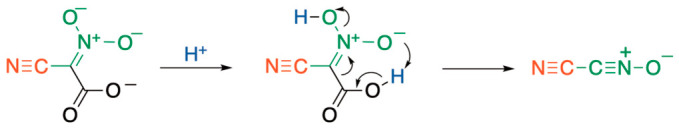
A speculation to generate cyano-functionalized nitrile oxide.

As discussed in Section 2.4, the practical application of (cyano)nitrile oxide has been severely limited by its extreme instability and the lack of suitable precursor systems. A practical solution to this problem has been realized through the use of cyano-*aci*-nitroacetate, which is readily obtained by ring opening of PyIOx. Because this intermediate is the dianion of α-nitro(cyano)acetic acid, protonation is expected to trigger decarboxylation and dehydration, thereby generating (cyano)nitrile oxide in situ (Scheme 31). This strategy provides an attractive alternative to the direct generation of this highly unstable nitrile oxide.

**Scheme 32:**
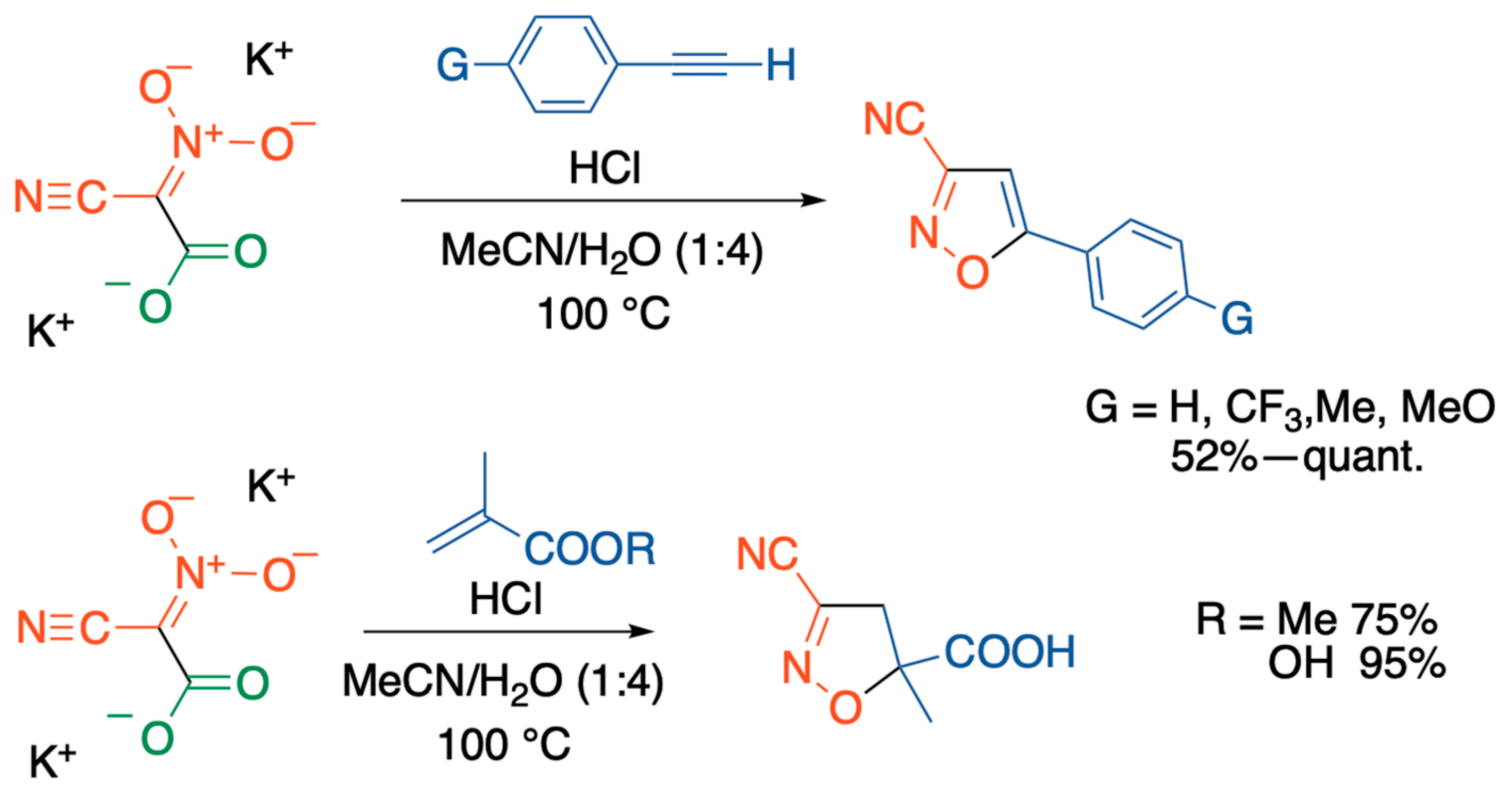
Synthesis of cyano-substituted isoxazol(in)es.

When cyano-*aci*-nitroacetate is heated with alkynes or alkenes under acidic conditions, efficient cycloaddition occurs to afford the corresponding cyano-substituted isoxazoles and isoxazolines, respectively (Scheme 32) [107]. Under the reaction conditions, ester functionalities are not sufficiently stable and are hydrolyzed to afford [(cyano)isoxazoline]carboxylic acids. Nevertheless, this observation demonstrates that α,β-unsaturated carboxylic acids, which are generally unsuitable dipolarophiles for conventional nitrile oxide chemistry, can participate directly in the cycloaddition. Consistent with this interpretation, methacrylic acid undergoes smooth cycloaddition to furnish the identical product (Scheme 32), further expanding the scope of compatible dipolarophiles [107].

**Scheme 33:**
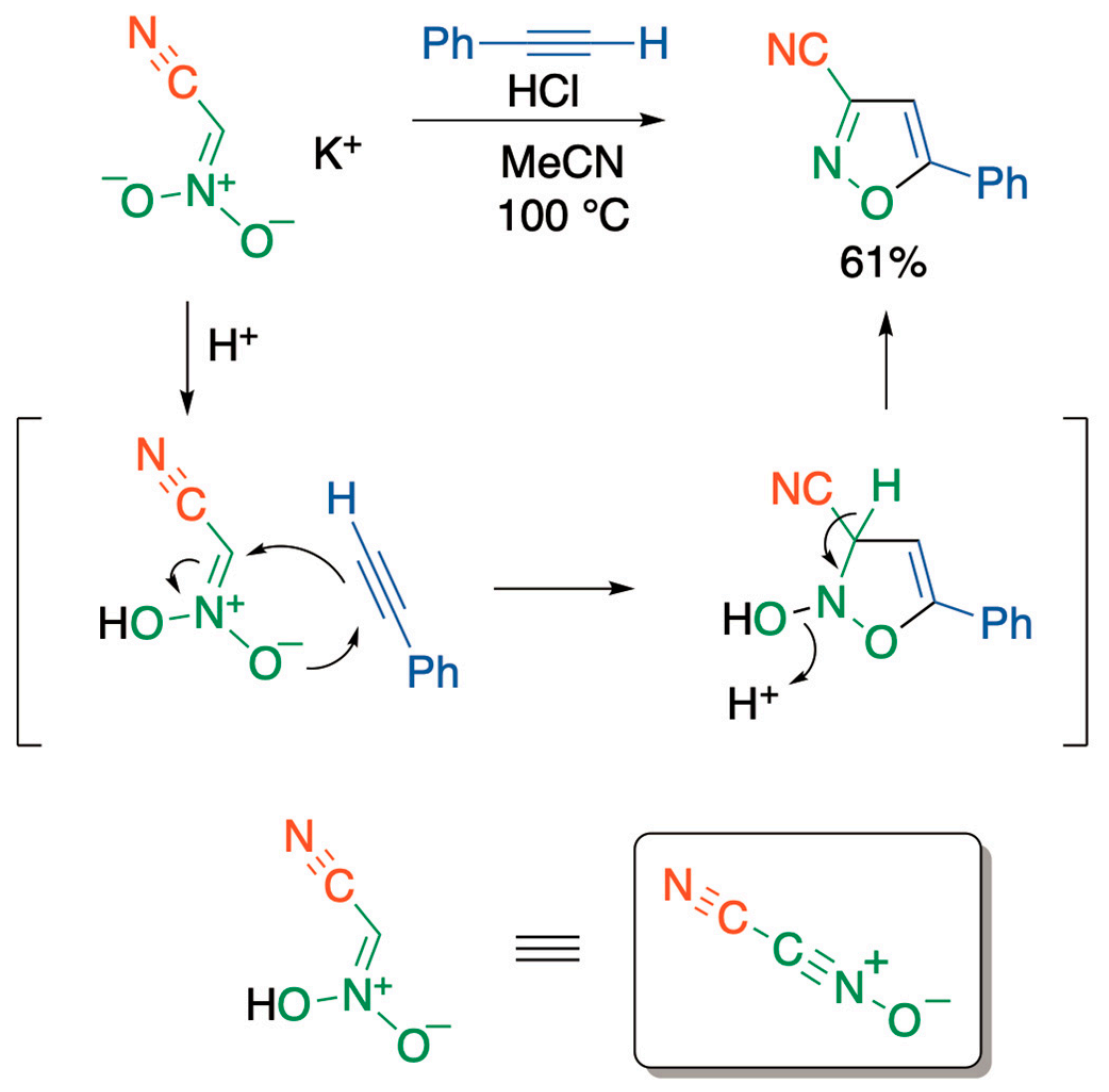
Synthetic equivalent of (cyano)nitrile oxide.

Although the above results are consistent with the intermediacy of (cyano)nitrile oxide, its exceptionally high instability makes it unlikely that the free nitrile oxide persists for any significant time under the acidic aqueous reaction conditions. Mechanistic insight was obtained from experiments using the decarboxylated potassium salt of nitroacetonitrile. Treatment of this independently prepared intermediate with dipolarophiles afforded the same cycloadducts as those obtained from cyano-*aci*-nitroacetate (Scheme 33), indicating that the actual 1,3-dipole is (cyano)nitronate, rather than the free nitrile oxide itself. Accordingly, (cyano)nitronate should be regarded as a synthetic equivalent of (cyano)nitrile oxide, reproducing its characteristic cycloaddition reactivity without requiring direct generation of the highly unstable nitrile oxide.

This concept represents a significant advance in the chemistry of functionalized nitrile oxides. Rather than relying on the transient existence of (cyano)nitrile oxide, the desired cycloaddition chemistry can be achieved through a stable and readily accessible nitronate intermediate. Consequently, this strategy overcomes one of the principal limitations discussed in Section 2.4 and establishes a practical synthetic platform for the preparation of cyano-substituted isoxazole and isoxazoline derivatives.

## 4. Conclusions

This review has summarized recent advances in the generation of functionalized nitrile oxides and the development of their synthetic equivalents. Although numerous methods for generating nitrile oxides have been reported, the direct preparation of functionalized nitrile oxides remains challenging because suitable precursor molecules are often difficult to access and many functional groups are incompatible with the reaction conditions required for nitrile oxide generation. Recent developments have demonstrated that these longstanding limitations can be overcome through the design of practical precursor systems and synthetic equivalents.

Among these advances, MeIOx has emerged as a particularly versatile precursor to (*N*-methylcarbamoyl)nitrile oxide, which can be generated under exceptionally mild aqueous conditions. The resulting nitrile oxide undergoes efficient 1,3-dipolar cycloaddition with a broad range of dipolarophiles, including alkenes, alkynes, nitriles, and 1,3-dicarbonyl compounds, providing straightforward access to structurally diverse isoxazoles, isoxazolines, and 1,2,4-oxadiazoles. Moreover, post-cycloaddition transformation of the *N*-methylcarbamoyl group into carboxy, ester, amide, acyl, and formyl functionalities greatly expands the synthetic utility of this methodology, effectively enabling MeIOx to function as a synthetic equivalent of a wide variety of functionalized nitrile oxides. Likewise, cyano-*aci*-nitroacetate, generated by ring opening of PyIOx, serves as a practical synthetic equivalent of (cyano)nitrile oxide, providing access to cyano-substituted isoxazole(in)es without requiring direct generation of this highly unstable nitrile oxide.

An additional advantage of the products obtained by these methods is the presence of the isoxazole N–O bond, which can be readily cleaved to afford a diverse range of highly functionalized molecular frameworks. Consequently, the strategies described in this review provide efficient access to multifunctional compounds that are difficult or impossible to prepare by conventional synthetic methods.

The development of practical precursors and synthetic equivalents has transformed functionalized nitrile oxide chemistry from a field largely limited by precursor availability into a versatile platform for heterocycle synthesis and molecular functionalization. Continued advances in this area are expected to further broaden the scope of functionalized nitrile oxides and accelerate the discovery of new bioactive molecules, functional materials, and advanced molecular architectures.

## Data Availability

Dataset available on request from the authors.
